# Identification of key enzymes responsible for protolimonoid biosynthesis in plants: Opening the door to azadirachtin production

**DOI:** 10.1073/pnas.1906083116

**Published:** 2019-08-01

**Authors:** Hannah Hodgson, Ricardo De La Peña, Michael J. Stephenson, Ramesha Thimmappa, Jason L. Vincent, Elizabeth S. Sattely, Anne Osbourn

**Affiliations:** ^a^Department of Metabolic Biology, John Innes Centre, Norwich Research Park, Norwich NR4 7UH, Norfolk, United Kingdom;; ^b^Department of Chemical Engineering, Stanford University, Stanford, CA 94305;; ^c^Jealott’s Hill International Research Centre, Syngenta Ltd., Bracknell RG42 6EY, Berkshire, United Kingdom;; ^d^Howard Hughes Medical Institute, Stanford University, Stanford, CA 94305

**Keywords:** natural products, limonoids, terpenes, insecticides, neem

## Abstract

Limonoids are natural products made by plants belonging to the Meliaceae (Mahogany) and Rutaceae (Citrus) families. They are well known for their insecticidal activity, contribution to bitterness in citrus fruits, and potential pharmaceutical properties. The best known limonoid insecticide is azadirachtin, produced by the neem tree (*Azadirachta indica*). Despite intensive investigation of limonoids over the last half century, the route of limonoid biosynthesis remains unknown. Limonoids are classified as tetranortriterpenes because the prototypical 26-carbon limonoid scaffold is postulated to be formed from a 30-carbon triterpene scaffold by loss of 4 carbons with associated furan ring formation, by an as yet unknown mechanism. Here we have mined genome and transcriptome sequence resources for 3 diverse limonoid-producing species (*A. indica*, *Melia azedarach*, and *Citrus sinensis*) to elucidate the early steps in limonoid biosynthesis. We identify an oxidosqualene cyclase able to produce the potential 30-carbon triterpene scaffold precursor tirucalla-7,24-dien-3β-ol from each of the 3 species. We further identify coexpressed cytochrome P450 enzymes from *M. azedarach* (MaCYP71CD2 and MaCYP71BQ5) and *C. sinensis* (CsCYP71CD1 and CsCYP71BQ4) that are capable of 3 oxidations of tirucalla-7,24-dien-3β-ol, resulting in spontaneous hemiacetal ring formation and the production of the protolimonoid melianol. Our work reports the characterization of protolimonoid biosynthetic enzymes from different plant species and supports the notion of pathway conservation between both plant families. It further paves the way for engineering crop plants with enhanced insect resistance and producing high-value limonoids for pharmaceutical and other applications by expression in heterologous hosts.

## Significance

Limonoids are natural products made by members of the Meliaceae and Rutaceae families. Some limonoids (e.g., azadirachtin) are toxic to insects yet harmless to mammals. The use of limonoids in crop protection and other applications currently depends on extraction from limonoid-producing plants. Metabolic engineering offers opportunities to generate crop plants with enhanced insect resistance and also to produce high-value limonoids (e.g., for pharmaceutical use) by expression in heterologous hosts. However, to achieve this the enzymes responsible for limonoid biosynthesis must first be characterized. Here we identify 3 conserved enzymes responsible for the biosynthesis of the protolimonoid melianol, a precursor to limonoids, from *Melia azedarach* and *Citrus sinensis*, so paving the way for limonoid metabolic engineering and diversification.

**Fig. 1.**
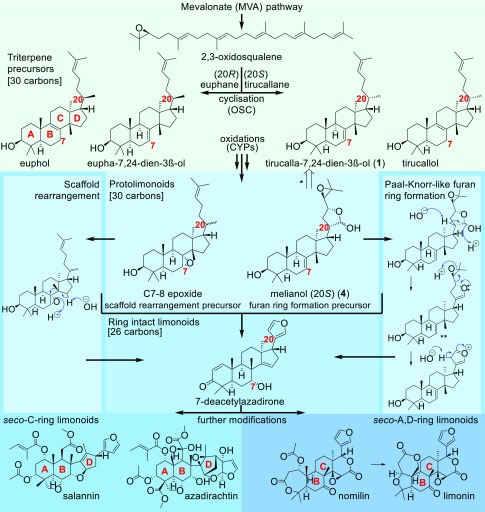
Hypothetical route of limonoid biosynthesis. Predictions of the major biosynthetic steps required for the biosynthesis of limonoids. The triterpene precursor 2,3-oxidosqualene is proposed to be cyclized to an unconfirmed tetracyclic triterpene scaffold. The structure of ring-intact limonoids implicates a tetracyclic triterpene precursor of either the euphane (20*R*) or tirucallane (20*S*) type. Retrosynthetic discrimination between these 2 side chains is impossible based on limonoid structures, because the formation of the furan ring eradicates any remnants of the precursor’s C20 stereochemistry. However, predictions can be made based on the immediate precursors of limonoids (protolimonoids); for instance, the C20 carbon of melianol (**4**) has been assigned (although not yet confirmed by X-ray crystallography) as the *S* configuration which implies a tirucallane precursor (60). Further, the C7-8 alkene of certain protolimonoids suggests the most likely triterpene precursor is in fact tirucalla-7,24-dien-3β-ol (**1**), as indicated by the retrosynthetic arrow (*), rather than tirucallol itself. Biosynthesis of limonoids from triterpene scaffolds is predicted to occur through protolimonoid structures such as melianol (**4**) and requires 2 major biosynthetic steps: scaffold rearrangements and furan ring formation accompanied by loss of 4 carbons. Scaffold rearrangement is proposed to be initiated by epoxidation of the C7 double bond (C7-8 epoxide) and furan ring formation could feasibly be initiated through oxidation and cyclization of the C20 tail (melianol) (**4**). The diversity of isolated protolimonoid structures has led to different predictions of the order of these 2 events (2, 61). The hemiacetal side chains of isolated protolimonoids such as melianol (**4**) suggest a Paal-Knorr-like (51, 52) route to the furan ring of limonoids. Isolation of nimbocinone from *A. indica* (*SI Appendix*, Fig. S1) (62), a feasible degradation product of this route (**), further supports conversion of protolimonoids to ring-intact limonoids through this mechanism. The ring-intact 7-deacetylazadirone has been isolated from both Meliaceae (63) and Rutaceae (64) species. Numerous further chemical transformations are required for the formation of *seco*-ring limonoid derivatives. In the Rutaceae, radioactive [^14^C]-labeling experiments in *C. limon* (lemon) have helped to delineate the late stages of the pathway, proving that nomilin can be biosynthesised in the stem and converted into other limonoids such as limonin and obacunone (*SI Appendix*, Fig. S1) elsewhere in the plant (65–67).

Limonoids are a diverse class of plant natural products. The basic limonoid scaffold has 26 carbon atoms (C26). Limonoids are classified as tetranortriterpenes because their prototypical structure is a tetracyclic triterpene scaffold (C30) which has lost 4 carbons during furan ring formation (1) (Fig. 1). The immediate precursors to limonoids (i.e., the C30 tetracyclic triterpenes preceding the loss of 4 carbons) are known as protolimonoids. Limonoids are heavily oxygenated and can exist either as simple ring-intact structures or as highly modified *seco*-ring derivatives (2) (Fig. 1). Limonoid production is largely confined to specific families within the Sapindales order (Meliaceae, Rutaceae, and to a lesser extent the Simaroubaceae) (3, 4).

Rutaceae limonoids have historically been studied because they are partially responsible for bitterness in citrus fruit. They have also been reported to have medicinal activities and so are of interest as potential pharmaceuticals. For example, 2 Rutaceae limonoids from citrus, limonin (5) and nomilin (6), show dose-dependent inhibition of HIV-1 viral replication in peripheral blood mononuclear cells at EC_50_ values of ∼50 to 60 μM (7). Further, the bioavailability of limonin glucoside (which is metabolized to limonin) has been observed in human trials (8). Around 50 limonoid aglycones have been reported from the Rutaceae, primarily with *seco*-A,D-ring structures (3, 4, 9) (Fig. 1). In contrast, the Meliaceae (Mahogany) are known to produce around 1,500 structurally diverse limonoids, of which the *seco-*C-ring limonoids are the most dominant (2, 4). *Seco*-C-ring limonoids (e.g., salannin and azadirachtin; Fig. 1) are generally regarded as particular to the Meliaceae and are of interest because of their antiinsect activity (2). Azadirachtin (isolated from *Azadirachta indica*) is particularly renowned because of its potent insect antifeedant activity. Insecticidal effects of azadirachtin have been observed at concentrations of 1 to 10 ppm (1). Other reported features make azadirachtin suitable for crop protection, such as systemic uptake, degradability, and low toxicity to mammals, birds, fish, and beneficial insects (1, 10). Extracts from *A. indica* seeds (which contain high quantities of azadirachtin) have a long history of traditional and commercial (e.g., NeemAzal-T/S, Trifolio-M GmbH) use in crop protection. Azadirachtin has a highly complex structure (Fig. 1) (11–14). Although the total chemical synthesis of this limonoid was reported in 2007, this represented the culmination of a 22-y endeavor (15) involving 71 steps and with 0.00015% total yield. Chemical synthesis of azadirachtin is therefore not practical for production on an industrial scale. Similarly, chemical synthesis of Rutaceae limonoids such as limonin (achieved in 35 steps from geraniol) (16) is also unlikely to be commercially viable. Therefore, at present the use of *seco*-C-ring Meliaceae limonoids for crop protection relies on extraction of *A. indica* seeds (1). Similarly, the potential health benefits of Rutaceae limonoids remain restricted to dietary consumption (17).

The involvement of the mevalonate (MVA) pathway in limonoid biosynthesis has been demonstrated by feeding experiments with ^14^C-mevalonate and ^13^C-glucose in *A. indica* plants and cell cultures, respectively (18, 19). The MVA pathway supplies the generic triterpene precursor 2,3-oxidosqualene, which can be cyclized to a variety of different triterpene scaffolds, a process initiated and controlled by enzymes known as oxidosqualene cyclases (OSCs) (20). Two OSC sequences have previously been identified in *A. indica*, but neither of the products of these genes has been functionally characterized (21). Additionally, an OSC has been identified in *Citrus grandis* and implicated in limonoid biosynthesis by viral-induced gene silencing (22). However, the *C. grandis* OSC is a close homolog to characterized lanosterol synthases (20), which would make involvement in limonoid biosynthesis unlikely. Thus, although speculation of potential biosynthetic routes is possible based on limonoid and protolimonoid structures (Fig. 1), the nature of the triterpene scaffold implicated in limonoid biosynthesis remains unknown.

The predicted route of limonoid biosynthesis beyond initial triterpene scaffold generation remains entirely speculative. In triterpene biosynthesis, oxidosqualene cyclization is commonly followed by oxidation, performed by cytochrome P450s (CYPs) (20). Several CYP sequences identified in *A. indica* and *C. grandis* have been implicated in limonoid biosynthesis based on expression profiling, in silico docking modeling, and phylogenetic analysis (21–26). However, these CYPs have not been functionally characterized and predictions of their activity are problematic without an understanding of the nature of the triterpene scaffold that they would act on. The only limonoid biosynthetic enzyme whose function has been confirmed by recombinant expression is a limonoid UDP‐glucosyltransferase from *Citrus unshiu*, which produces limonin-17‐β‐D‐glucopyranoside (27). The lack of enzymatic characterization is not due to an absence of genetic information as there is a wealth of sequencing data from limonoid-producing species of the Meliaceae (21, 23, 24, 26, 28–33) and Rutaceae families (https://www.citrusgenomedb.org/).

Here we have utilized available genome and transcriptome resources for Meliaceae (21, 28, 31, 32) and Rutaceae (34) species to begin to elucidate the biosynthetic pathway to structurally complex and important limonoids such as azadirachtin. Phylogenetic analysis, gene expression analysis, and metabolite profiling have been used to identify candidate OSCs and CYPs from *Melia azedarach* and *Citrus sinensis*. Functional characterization of candidate genes by heterologous expression in *Saccharomyces cerevisiae* or transient expression in *Nicotiana benthamiana* has led to the identification of 3 enzymes from *M. azedarach* that together are capable of biosynthesis of the 30C protolimonoid, melianol. Characterization of the corresponding 3 *C. sinensis* homologs supports the notion of conserved initial biosynthesis for limonoids in Meliaceae and Rutaceae species. Our results represent the characterization of biosynthetic genes involved in protolimonoid formation.

## Results and Discussion

### Characterization of OSCs Catalyzing the Formation of Tirucall-7,24-dien-3β-ol from 3 Limonoid-Producing Species.

**Fig. 2.**
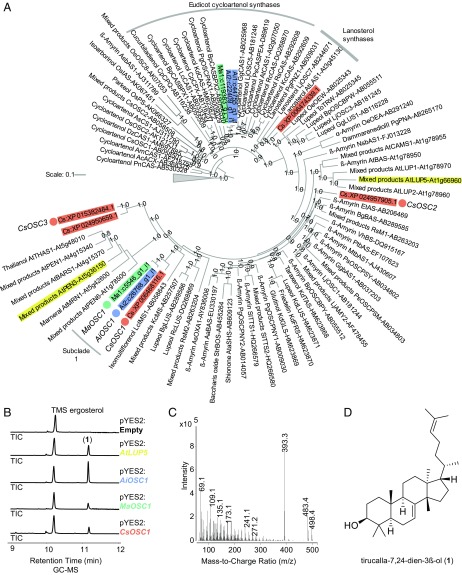
Identification and characterization of OSCs from limonoid-producing species. (*A*) Phylogenetic tree of candidate OSCs from *A. indica* (blue), *M. azedarach* (green), and *C. sinensis* (orange). Functionally characterized OSCs from other plant species (20) are included, with the 2 previously characterized tirucalla-7,24-dien-3β-ol synthases from *A. thaliana* (*AtLUP5* and *AtPEN3*) highlighted (yellow). Human and prokaryotic OSC sequences used as an outgroup are represented by the gray triangle. Candidate OSCs chosen for further analysis are indicated (circles). The phylogenetic tree was constructed by FastTree V2.1.7 (68) and formatted using iTOL (69). Local support values from FastTree Shimodaira-Hasegawa (SH) test (between 0.6 and 1.0) are indicated at nodes, and scale bar depicts estimated number of amino acid substitutions per site. (*B*) GC-MS total ion chromatograms of derivatized extracts from yeast strains expressing candidate OSCs. Traces for the empty vector (pYES2) and strains expressing the candidates AiOSC1 (blue), MaOSC1 (green), CsOSC1 (orange), and the previously characterized AtLUP5 (yellow) are shown. (*C*) GC-MS mass spectra of TMS-tirucalla-7,24-dien-3β-ol (**1**). (*D*) Confirmation of the structure of the cyclization product generated by AiOSC1 as tirucalla-7,24-dien-3β-ol (**1**) by NMR (*SI Appendix*, Table S3). Traces, mass spectra, and product structure for CsOSC2 and CsOSC3 are given (*SI Appendix*, Figs. S2 and S3).

Four sequence resources were used to search for candidate genes implicated in limonoid biosynthesis: a *C. sinensis* var. Valencia genome annotation downloaded from National Center for Biotechnology Information (NCBI) (34); 2 *A. indica* transcriptomes that we assembled from raw RNAseq data downloaded from NCBI-Sequence Read Archive (SRA), using the Trinity de novo assembler (21, 28, 31); and a *M. azedarach* transcriptome that we assembled using the same method (32) (*SI Appendix*, Table S1). The protein sequences of 83 previously characterized OSCs (20) were used as a BLAST+ query. Hits were filtered based on predicted protein sequence length and presence of a conserved triterpene synthase motif (35). Phylogenetic analysis revealed that of the 10 candidate OSCs identified, 3 grouped with a conserved clade containing characterized cycloartenol synthases, and a fourth with lanosterol synthases (Fig. 2*A*). These OSCs are therefore likely to have functions in sterol biosynthesis rather than in limonoid biosynthesis (20). The remaining candidates fell into other more diverse triterpene OSC clades and so were more likely to have roles in specialized metabolism. Three of these were from *C. sinensis*, 1 from *A. indica*, and another from *M. azedarach*. One of the *C. sinensis* OSCs formed a tight subclade (subclade 1, Shimodaira-Hasegawa [SH] local support value of 1) with the latter 2 Meliaceae candidates (Fig. 2*A*), making these the most promising candidates for limonoid biosynthesis.

Functional characterization of these 3 OSC candidates (named AiOSC1, MaOSC1, and CsOSC1) (Fig. 2*A*) was performed by expression in *S. cerevisiae* strain GIL77 (*SI Appendix*, Table S2). A single major product with the same retention time was detected when extracts of yeast strains expressing each of the 3 OSCs were analyzed by gas chromatography-mass spectrometry (GC-MS). This product was tentatively identified as tirucalla-7,24-dien-3β-ol (**1**; boldface nos. indicate structures found in Figs. 2 *B* and *D* and 5 *B*–*E*) in all 3 cases, based on its mass spectrum. Comparison of the retention time and mass spectra of the product with that of the multifunctional OSC AtLUP5 (from *Arabidopsis thaliana*), which produces tirucalla-7,24-dien-3β-ol (**1**) as part of its product profile, was consistent with this (Fig. 2 *B* and *C*) (20, 36, 37). We next isolated and purified the AiOSC1 product and confirmed its structure as tirucalla-7,24-dien-3β-ol (**1**) by NMR (Fig. 2*D* and *SI Appendix*, Table S3). The 2 other phylogenetically distinct OSCs from *C. sinensis* (CsOSC2 and CsOSC3, indicated in Fig. 2*A*) were also expressed in yeast and found to make different products, which were identified as β-amyrin and lupeol based on comparison with standards (*SI Appendix*, Figs. S2 and S3 and Table S2).

Although the sequences of the 3 previously reported uncharacterized putative OSCs (2 from *A. indica* and 1 from *C. grandis*) (21, 22) have not been deposited in publicly available databases, they appear to be phylogenetically distinct from the 3 tirucalla-7,24-dien-3β-ol synthases characterized here (based on phylogeny and closest reported BLAST hits). They are also clearly distinct from AtLUP5. However, another previously characterized multifunctional OSC (AtPEN3) from *A. thaliana* that produces tirucalla-7,24-dien-3β-ol (20, 36, 37) is located in a neighboring subclade in the tree (Fig. 2*A*).

### Expression of *AiOSC1* and *MaOSC1* in Limonoid-Accumulating Tissues.

**Fig. 3.**
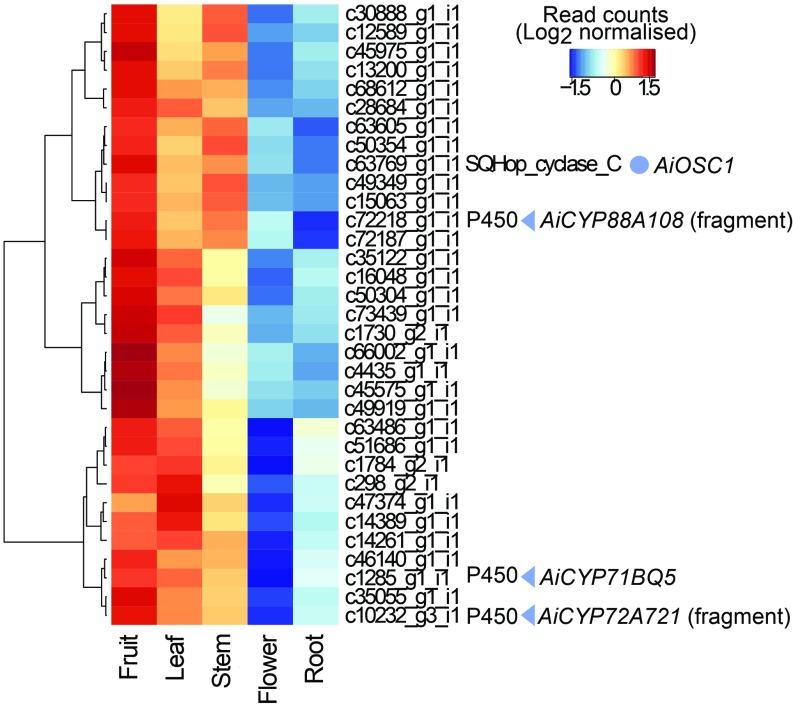
Expression patterns of *AiOSC1* and other coexpressed genes in *A. indica*. A heatmap of a subset of differentially expressed (*P* < 0.05) genes with similar expression patterns to *AiOSC1* (blue circle) across flower, root, fruit, and leaf tissues of *A. indica* are shown. Raw RNAseq reads (28, 31) were aligned to a Trinity-assembled transcriptome of the same dataset. Read counts were normalized to library size and log_2_ transformed. Values depicted are scaled by row (gene) to emphasize differences across tissues. The Pfam identifier for relevant predicted gene is included next to the contig number. Genes with no structural (Augustus) or functional (Pfam) annotations have been excluded. CYP candidates *AiCYP71BQ5*, *AiCYP72A721*, and *AiCYP88A108* (blue triangles) are indicated with the latter 2 being considered gene fragments (<300 amino acids).

Differential gene expression analysis and hierarchal clustering was performed using available *A. indica* RNAseq data (28, 31). *AiOSC1* showed highest expression in the fruit (Fig. 3), consistent with a previous report of high levels of the ring-intact limonoids azadiradione and epoxyazadiradione (*SI Appendix*, Fig. S1) in *A. indica* in this organ (21). Other genes that were highly coexpressed with *AiOSC1* included 3 predicted CYP sequences. These were named by the Cytochrome P450 Nomenclature Committee following established convention (38) as *AiCYP71BQ5*, *AiCYP72A721*, and *AiCYP88A108* (Fig. 3). These coexpressed CYPs are implicated as potential candidates for oxidation of the tirucalla-7,24-dien-3β-ol (**1**) scaffold produced by *AiOSC1*.

**Fig. 4.**
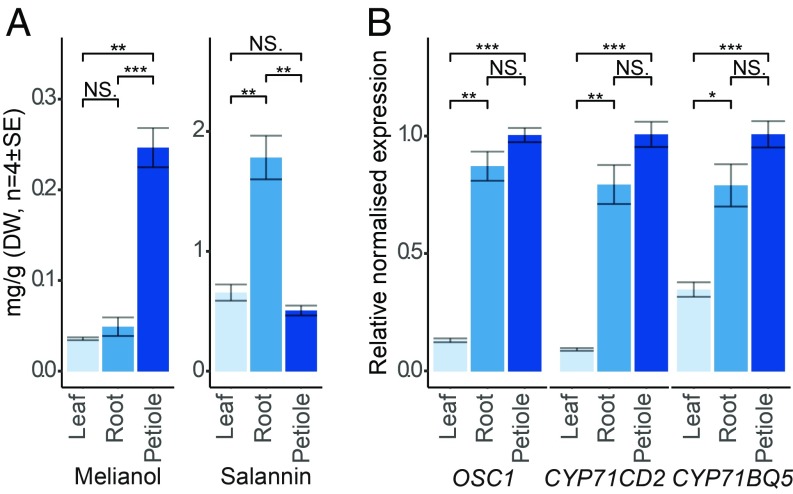
Accumulation of melianol and salannin and expression of *MaOSC1*, *MaCYP71CD2*, and *MaCYP71BQ5* in *M. azedarach*. (*A*) Estimated concentrations (mg/g DW, *n* = 4 ± SE) of the protolimonoid melianol (**4**) and the *seco-*C-ring limonoid salannin in extracts from *M. azedarach* leaf, root, and petiole tissue. (*B*) Normalized expression of *MaOSC1*, *MaCYP71CD2*, *and MaCYP71BQ5* relative to *Maß-actin* in RNA from leaves, roots, and petioles of *M. azedarach* by qRT-PCR. Relative expression levels were calculated using the ΔΔCq method (58) (*n* = 4 ± SE). T-test significance values are indicated: not significant (NS), *P* value ≤ 0.05 (*), 0.01 (**), or ≤ 0.001 (***).

Unlike *A. indica*, the spatial occurrence of limonoids within other Meliaceae species has not been investigated. *M. azedarach*, a close relative of *A. indica* (39), is the second most prolific limonoid-producing species with 109 limonoid structures reported, including *seco*-C-ring limonoids of the azadirachtin and meliacarpin class (2, 4). We therefore investigated the levels of melianol and salannin in extracts from the leaves, roots, and petioles of young (∼12 mo) *M. azedarach* plants. Azadirachtin was not detected in our analyses, consistent with an earlier investigation (32). Melianol accumulation was significantly higher in extracts from the petiole compared with root and leaf tissue, while salannin accumulation was highest in the roots (Fig. 4*A*). The relative expression levels of *MaOSC1* in available *M. azedarach* tissues were significantly higher in the tissues identified as having the highest accumulation of melianol and salannin, the petiole and root tissues, respectively (Fig. 4*B*).

The expression of *A. indica* and *M. azedarach AiOSC1* and *MaOSC1* in tissues that accumulate limonoids and the lack of an alternative candidate OSC shared between these limonoid-producing species together suggest that these OSCs may catalyze the first step in limonoid biosynthesis. The characterization of these 2 OSCs along with CsOSC1 as tirucalla-7,24-dien-3β-ol synthases supports the hypothesis that tirucalla-7,24-dien-3β-ol (**1**) is the triterpene precursor of limonoids in these species. Our finding is in contrast to an earlier *A. indica* study (40) which reported a greater relative incorporation of [^3^H]-labeled euphol into the *seco*-C-ring limonoid nimbolide (*SI Appendix*, Fig. S1), compared with the tirucallanes or other euphanes. However, this earlier report employed inconsistent methods of precursor labeling and used wet leaf weight in calculations of relative incorporation, and so the results may be unreliable.

### Identification of 2 Cytochrome P450 Enzymes from *M. azedarach* That Together Convert Tirucall-7,24-dien-3β-ol to Melianol.

**Fig. 5.**
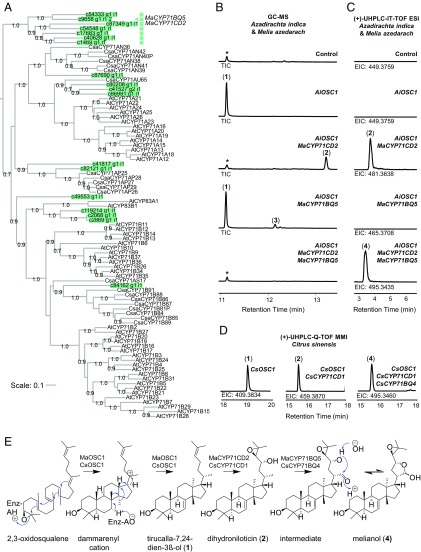
Identification and functional analysis of cytochrome P450 enzymes capable of melianol biosynthesis. (*A*) A subset of a larger phylogenetic tree (*SI Appendix*, Fig. S4) showing the CYP71 family. Candidate CYPs from *M. azedarach* (green) and previously identified CYPs from *A. thaliana* (http://www.p450.kvl.dk) and *C. sativus* (http://drnelson.uthsc.edu/cytochromeP450.html) (black) are included. Candidate CYPs selected for cloning (*SI Appendix*, Table S4) were identified by homology to *A. indica* candidate CYPs identified as coexpressed with *AiOSC1* (triangle) or occurrence in a unique CYP71 subclade lacking close homologs from *A. thaliana* or *C. sativus* (squares). The phylogenetic tree was constructed by FastTree V2.1.7 (68) and formatted using iTOL (69). Local support values from FastTree Shimodaira-Hasegawa (SH) test (between 0.6 and 1.0) are indicated at nodes, and scale bar depicts estimated number of amino acid substitutions per site. Data from the *Arabidopsis* Cytochrome P450, Cytochrome b_5_, P450 Reductase, β-Glucosidase, and Glycosyltransferase Site, and from ref. 70. GC-MS total ion chromatograms (*B*) and LC-MS electrospray ionization (ESI) extracted ion chromatograms (*C*) of triterpene extracts from agroinfiltrated *N. benthamiana* leaves expressing *A. indica* and *M. azedarach* candidate genes in the pEAQ-*HT*-DEST1 vector. (*D*) LC-MS multimode ionization (MMI) extracted ion chromatograms of triterpene extracts from agroinfiltrated *N. benthamiana* leaves expressing *C. sinensis* candidate genes in the pEAQ-*HT*-DEST1 vector. Mass spectra for products are shown in *SI Appendix*, Figs. S5–S7. The peak marked with an asterisk is an endogenous *N. benthamiana* peak, not tirucalla-7,24-dien-3β-ol (**1**) (*SI Appendix*, Fig. S8). (*E*) Proposed pathway of melianol (**4**) biosynthesis in *M. azedarach*. NMR confirmation of all structures can be found in *SI Appendix*, Tables S3 and S5–S7.

To identify candidate CYPs that may be capable of oxidizing the tirucalla-7,24-dien-3β-ol scaffold, the protein sequences of 235 *A. thaliana* CYPs (downloaded from http://www.p450.kvl.dk) were used to BLAST+ (41) search the Trinity-assembled *M. azedarach* transcriptome. Phylogenetic comparison with *A. thaliana* and *Cucumis sativus* (http://drnelson.uthsc.edu/cytochromeP450.html) CYPs (the latter included as an additional dicotyledon species for which full genomic complement of CYPs have been assigned) revealed that while most of the 103 candidate *M. azedarach* CYPs were dispersed throughout the tree, a discrete subclade of 7 *M. azedarach* CYPs (SH local support value of 1) was phylogenetically distinct from all *A. thaliana* and *C. sativus* candidates (*SI Appendix*, Fig. S4 and Fig. 5*A*). This subclade sits within the largest CYP family in plants (CYP71), which is known for taxa-specific subfamily blooms and includes CYPs with characterized roles in secondary metabolism (42). Further the subclade includes a candidate CYP (*MaCYP71BQ5*) which is an ortholog of *AiCYP71BQ5*. *AiCYP71BQ5* is coexpressed with *AiOSC1* in *A. indica* (Fig. 3). We selected a total of 9 *M. azedarach* candidate CYPs (*SI Appendix*, Table S4)—the 7 from the CYP71 subclade and a further 2 that were homologous to 2 other *A. indica* CYPs that were coexpressed with *AiOSC1*—for further analysis. Two of the candidates, *MaCYP71CD2* and *MaCYP71BQ5*, share a similar expression pattern to *MaOSC1* (Fig. 4*B*).

To determine the ability of the candidate CYPs to modify the tirucalla-7,24-dien-3β-ol scaffold (**1**), functional analysis was performed by transient coexpression with *AiOSC1* in *N. benthamiana* (43, 44). Expression of *AiOSC1* gave the expected product, tirucalla-7,24-dien-3β-ol (Fig. 5B), consistent with previous expression in yeast (Fig. 2 *B* and *C*). Coexpression of *AiOSC1* and *MaCYP71CD2* resulted in consumption of tirucalla-7,24-dien-3β-ol (**1**) and generation of a new product with a derivatized mass of 602.6 determined by GC-MS and an adduct of 481.365 determined by liquid chromatography (LC)-MS (**2**) (Fig. 5 *B* and *C* and *SI Appendix*, Figs. S5 and S6). This suggested that 2 oxidations of tirucalla-7,24-dien-3β-ol could have been performed by MaCYP71CD2, which could feasibly include a hydroxylation and a conversion of an alkene to either an epoxide or ketone. In contrast, coexpression of *AiOSC1* and *MaCYP71BQ5* resulted in partial consumption of tirucalla-7,24-dien-3β-ol (**1**) and production of a new product (**3**) (Fig. 5*B*). This new product (**3**) had a derivatized mass of 498.4 as determined by GC-MS (*SI Appendix*, Fig. S5) which could suggest a single hydroxylation of tirucalla-7,24-dien-3β-ol. When *AiOSC1* was coexpressed with both *MaCYP71CD2* and *MaCYP71BQ5*, tirucalla-7,24-dien-3β-ol was completely consumed and a new product (**4**) detectable by LC-MS with an adduct of 495.344 was observed (Fig. 5 *B* and *C* and *SI Appendix*, Fig. S6). The lack of detectable (**2**) and (**3**) suggests that these CYPs work sequentially to form (**4**). MaCYP71CD2 may act first due to greater efficiency of consumption of tirucalla-7,24-dien-3β-ol (**1**) than observed for MaCYP71BQ5 (Fig. 5*B*).

We next carried out large-scale coexpression, purified (**2**), (**3**), and (**4**), and determined their structures by NMR (Fig. 5*E* and *SI Appendix*, Tables S5–S7). Our structural analysis revealed that MaCYP71CD2 introduces a secondary alcohol at C23 and an epoxide at the C24-25 alkene of tirucalla-7,24-dien-3β-ol to give the previously isolated compound dihydroniloticin (**2**), a postulated protolimonoid. Dihydroniloticin and its C3 ketone, niloticin, have previously been isolated on multiple occasions from Meliaceae, Rutaceae, and Simaroubaceae species. Several structures with C23 oxidation only (lacking the C24 epoxide) have also been reported. However, the occurrence of niloticin-type structures with both the epoxidation and C23 oxidation are far more common (*SI Appendix*, Table S8). MaCYP71BQ5 introduces a primary alcohol at C21 of (**1**) to form the previously isolated compound tirucalla-7,24-dien-21,3β-diol (**3**), another postulated protolimonoid. Tirucalla-7,24-dien-21,3β-diol (**3**) has been isolated only once from an obscure member of the Simaroubaceae family (45), while a related structure with an aldehyde at C21, 3-oxotirucalla-7,24-dien-21-al, has been isolated a total of 4 times from members of Meliaceae, Rutaceae, and Simaroubaceae families (*SI Appendix*, Table S8). The mass adduct of the product of coexpression of AiOSC1 MaCYP71CD2, and MaCYP71BQ5 (**4**) does not correspond to the predicted product of MaCYP71CD2 and MaCYP71BQ5 acting together (tirucalla-7-ene-24,25-epoxy-3β,21,23-triol). It appears that the combination of introduction of a secondary alcohol at C23, epoxidation of the C24-25 alkene and oxidation at C21, performed by MaCYP71CD2 and MaCYP71BQ5, together causes spontaneous hemiacetal ring formation by nucleophilic attack of C21 to give the known protolimonoid melianol (**4**) (Fig. 5*E*). Although the structure isolated as the product of MaCYP71BQ5 contains a primary alcohol group at C21, the formation of melianol suggests MaCYP71BQ5 in fact produces an aldehyde at this position which would allow the formation of the melianol hemiacetal ring. Melianol exists as an epimeric mixture in solution (46), with the hemiacetal ring opening and reforming with 2 different stereochemistries at C21. Similar epimeric mixtures have been reported in other protolimonoids containing a hemiacetal ring structure such as turraeanthin (47) and melianone (48) (*SI Appendix*, Table S8). Although melianol (**4**) has only been isolated 8 times, its C3 ketone melianone has been isolated from a total of 18 species across the Meliaceae, Rutaceae, and Simaroubaceae families (*SI Appendix*, Table S8).

The previous isolation of the products generated by MaCYP71CD2 and MaCYP71BQ5 from members of all 3 limonoid-producing families of plants suggests that the biosynthesis of melianol could represent the initial stage of limonoid biosynthesis across the Sapindales order. Consistent with this, close homologs of *MaCYP71CD2* and *MaCYP71BQ5* are present in *A. indica* and *C. sinensis* (*SI Appendix*, Fig. S9). Functional analysis of *C. sinensis* homologs CsCYP71CD1 and CsCYP71BQ4 in *N. benthamiana* by LC-MS confirmed sequential production of (**2**) to (**4**) (Fig. 5*D*). Transcriptome mining of publicly available data from *C. sinensis* shows coexpression of these 2 CYPs and CsOSC1 (49). (*SI Appendix*, Table S9).

The pathway shown in Fig. 5*E* is the proposed pathway for melianol biosynthesis in *M. azedarach* and *C. sinensis*, based on the evidence presented here. Our work provides an example of the functional characterization of biosynthetic enzymes involved in protolimonoid biosynthesis. Together MaCYP71CD2 and MaCYP71BQ5 and homologs CsCYP71CD1 and CsCYP71BQ4 are capable of catalyzing the 3 oxygenations of tirucalla-7,24-dien-3β-ol required to induce hemiacetal ring formation, so affording the protolimonoid melianol. The identification of enzymes capable of initial ring formation on the side chain of tirucalla-7,24-dien-3β-ol are feasibly the starting process of furan ring formation (Fig. 1). Thus, the identification of these enzymes could imply that the order of chemical transformations in the subsequent conversion of protolimonoids to limonoids begins with furan ring formation (Fig. 1). The isolation of protolimonoid 7-deacetylbruceajavanin B (50), which possesses a hemiacetal ring and typical limonoid internal scaffold rearrangements (*SI Appendix*, Fig. S1) is counter to this, but could still support formation of a hemiacetal ring before scaffold rearrangements.

Limonoids are unusual in comparison with other characterized triterpene pathways in that they require extensive modifications to the triterpene scaffold itself (Fig. 1). The enzymes responsible for the next steps are therefore unprecedented in triterpene biosynthesis and may be challenging to identify. Nevertheless, the foundation that we have established now opens up opportunities to elucidate further pathway steps by coexpressing new candidate genes with the protolimonoid pathway steps characterized here. Biosynthetic access to melianol has opened the door to exploring the biosynthesis of the limonoids. While the downstream pathway steps are currently unknown, further development of the strategies employed here may in the future yield the 2 further enzymatic steps believed to be needed for scaffold rearrangement, and the 2 to 4 enzymes potentially required to complete the Paal-Knorr-like (51, 52) route to the furan ring (Fig. 1). Hydrolases, oxygenases, dehydratases, desaturases, and isomerases could all be involved in such transformations and therefore high-throughput screening of diverse enzyme classes will be required to elucidate the next biosynthetic steps. This would furnish the basal limonoid scaffold 7-deacetylazadirone (Fig. 1), which is likely to be a common intermediate shared by many members of this diverse class of natural products in both the Citrus and Meliaceae families.

### Conclusion.

Here we have characterized tirucalla-7,24-dien-3β-ol synthase OSCs (AiOSC1, MaOSC1, CsOSC1) from 3 limonoid-producing plant species (*A. indica*, *M. azedarach*, and *C. sinensis*). We have further identified 2 *M. azedarach* CYPs which together are capable of 3 oxidations of the tail of the tirucalla-7,24-dien-3β-ol (**1**), so causing spontaneous hemiacetal ring formation, to give the protolimonoid melianol (**4**). We then demonstrated evidence for conservation of early stage limonoid biosynthesis across the Meliaceae and Rutaceae families by replicating our results using *C. sinensis* homologs. Our study reports the characterization of the enzymes required for protolimonoid biosynthesis and will provide a foundation for elucidation of the downstream pathways for limonoid biosynthesis in the future.

## Materials and Methods

### *M. azedarach* Material.

A young (<1 y) *M. azedarach* plant was purchased from Crûg Farm Plants (UK) in summer 2016 and maintained in a John Innes Centre greenhouse (24 °C, 16 h light, grown in John Innes Cereal mix). The individual’s provenance is Chikugogawa Prefectural Natural Park (Japan). Seeds were collected by Crûg Farm Plants in autumn 2015 from an area of the park with no sampling restrictions. Confirmation that the material was out of scope of the Nagoya Protocol and Access and Benefit Sharing legislation was given by the National Focal Point of Japan.

### Transcriptome Assembly.

Raw RNAseq reads from 2 studies of *A. indica* (21, 28, 31) and 1 study of *M. azedarach* (32) were downloaded from NCBI-SRA (*SI Appendix*, Table S1). Within each dataset, tissues were pooled and a reference transcriptome was assembled using Trinity de novo assembler V.r0140717 (53) following a standard protocol (54) (*SI Appendix*, Table S1). For protein annotation Augustus V3.2.2 (55) was used in intron-less mode with an *A. thaliana* training model and untranslated region (UTR) identification turned off.

### Identification of Oxidosqualene Cyclases.

Protein sequences of 83 functionally characterized OSCs (20) were used as a query for identification of candidate OSCs by BLAST+ V2.7.1 (41) searches of Trinity-assembled transcriptomes (*SI Appendix*, Table S1) and a *C. sinensis* protein annotation (GCF_000317415) (34). Candidates were filtered based on the presence of the conserved triterpene synthase OSC motif (DCTAE) (35), length (between 700 and 1,000 amino acids), and prediction of a protein coding sequence (Augustus V3.2.2) (55). Five unique candidate OSCs were present in *C. sinensis* and 2 in both *M. azedarach* and *A. indica*. Protein sequences were aligned with MUSCLE V3.8.31 (56).

### Characterization of Candidate Oxidosqualene Cyclases.

Candidate OSC sequences from *C. sinensis* and *A. indica* were synthesized (Integrated DNA Technologies) in 2 fragments and recombined into the pYES2 vector (Thermo Fisher Scientific). *MaOSC1* was cloned from *M. azedarach* into pYES2, and the cloned gene for *AtLUP5*, a previously characterized OSC (36), was sourced through TAIR (stock: U16880). Details of cloning methods of OSC candidates (*SI Appendix*, Table S2) are described in *SI Appendix*. Candidate OSCs were expressed in the yeast strain GIL77 (MATa/**α** *gal2 hem3-6 erg7 ura3-176*) (57) in 10-mL cultures. Triterpenes were extracted in hexane after saponification and analyzed by GC-MS (*SI Appendix*). Purification of the AiOSC1 product is described in *SI Appendix*. In addition, candidate OSCs were cloned into pEAQ-*HT-*DEST1 (*SI Appendix*) to enable agroinfiltration of *N. benthamiana* which was performed as previously described (44).

### *A. indica* Differential Gene Expression Analysis.

Using raw RNAseq data and the corresponding Trinity-assembled transcriptome of *A. indica* (28, 31), differential gene expression analysis was performed to identify a subset of differentially expressed genes (*P* < 0.05). Within this subset, hierarchal clustering analysis identified a cluster of genes with similar expression patterns to *AiOSC1* (*SI Appendix*). HMMSCAN (European Molecular Biology Laboratory-European Bioinformatics Institute [EMBL-EBI]) was used to assign pFAM domains with an E-value of 1.

### *M. azedarach* Limonoid and Protolimonoid Quantification.

Freeze-dried *M. azedarach* material was weighed (∼10 mg) and homogenized using tungsten carbide beads (3 mm; Qiagen) with a TissueLyser (1,000 rpm, 1 min). Samples were extracted in 550 µL 100% methanol (10 µg/mL podophyllotoxin internal standard, Sigma-Aldrich) and agitated at 18 °C for 20 min. Supernatant (400 µL) was transferred and mixed with 140 µL ddH_2_O. Defatting was performed by addition of hexane (400 µL) and removal of the upper phase (300 µL) in duplicate. Remaining solvent was evaporated to dryness and extracts were resuspended in 100 µL of methanol. Spin-X centrifuge tube filters (pore size 0.22 µm, Corning Costar) were used to filter extracts by centrifugation. Eluate (50 µL) was diluted in 50 µL of methanol and transferred to a glass insert placed inside a glass autosampler vial for LC-MS analysis (*SI Appendix*). LCMSsolutions V3 (Shimadzu) was utilized to analyze chromatograms and for peak identification. The internal standard (podophyllotoxin) was used to calculate an estimated concentration of target compound in starting material. Azadirachtin (Sigma-Aldrich), salannin (Greyhound Chromatography), and melianol (**4**) (*SI Appendix*) standards were used to confirm retention times and mass adducts.

### Identification of Candidate Cytochrome P450s in *M. azedarach*.

Candidate CYPs were identified in *M. azedarach* transcriptome data by a BLAST+ V2.7.1 (41) search using *A. thaliana* CYP protein sequences (http://www.p450.kvl.dk) as a query. A total of 1,672 hits were identified with 103 representing unique, full-length (300 to 700 amino acids) protein coding (Augustus V3.2.2) (55) sequences with “cytochrome P450” Pfam annotations (HMMSCAN) (EMBL-EBI). Protein sequences of candidates were aligned with MUSCLE V3.8.31 (56) to CYP protein sequences from *A. thaliana* (http://www.p450.kvl.dk) and *C. sativus* (http://drnelson.uthsc.edu/cytochromeP450.html). CYP clades were determined based on previous phylogenetic studies (42). The full phylogenetic tree is provided in *SI Appendix*, Fig. S4. Protein sequences of the 103 CYP candidates from *M. azedarach* were used as a BLAST+ V2.7.1 (41) query to identify homologs in *A. indica* and *C. sinensis.* CYPs were named following convention by the Cytochrome P450 Nomenclature Committee (38).

### Relative Expression of Candidate Genes in *M. azedarach*.

In the absence of a *M. azedarach* genome sequence, intron spanning PCR primers were designed for *Maß-Actin*, *MaOSC1*, *MaCYP71CD2*, and *MaCYP71BQ5* (*SI Appendix*, Table S10) by assuming similar intron patterning to the closely related *A. indica* species and subsequent alignment to the closest homologs in the *A. indica* draft genome (PRJNA176672; AMWY00000000.1) (30). Lightcycler 480 SYBR Green I Master mix (Roche) was used for quantitative real-time PCR (qRT-PCR) performed on a CFX96 real-time system and a C1000 touch thermal cycler (Bio-Rad). R was used to calculate relative expression compared with *Maß-actin* using the ΔΔCq method (58).

### Functional Analysis of Candidate Cytochrome P450s in *N. benthamiana*.

Candidate CYPs from *M. azedarach* were expressed in *N. benthamiana* by agroinfiltration of *Agrobacteria tumefaciens* LLBA4404 strains transformed with pEAQ-*HT*-DEST1 constructs (59). Different combinations of strains were coinfiltrated to test combinations of genes. A feedback insensitive HMG CoA-reductase (*AstHMGR*) was included in addition to the candidates due to its proven ability to boost triterpene yield in this system (44). Candidate CYPs from *C. sinensis* were characterized in *A. tumefaciens* GV3103 strain harboring pEAQ-*HT*-DEST1 constructs coinfiltrated with HMG CoA-reductase from *A. thaliana*. Agroinfiltration and harvest of leaf discs was performed as described previously for combinatorial triterpene biosynthesis (44). The methods outlined for extraction of limonoids and protolimonoids from *M. azedarac*h were used for analysis of infiltrated *N. benthamiana* leaves. Hexane extracts from the defatting process were retained for GC-MS analysis. Extracts were analyzed by GC-MS or LC-MS depending on the polarity of products formed (*SI Appendix*). Extraction of limonoids and protolimonoids from infiltrated *N. benthamiana* leaves with candidate CYPs from *C. sinensis* is described in *SI Appendix*. Purification of CYP products is described in *SI Appendix*.

## Supplementary Material

Supplementary File
