# Supplementary material for: Identification of key enzymes responsible for protolimonoid biosynthesis in plants: Opening the door to azadirachtin production
Source: Proc Natl Acad Sci U S A. 2019 Aug 1;116(34):17096–104. doi: 10.1073/pnas.1906083116 (PMC6708365; doi:10.1073/pnas.1906083116)
Supplement: Supplementary File [file pnas.1906083116.sapp.pdf]

Supplementary Information

# Identification of key enzymes responsible for protolimonoid biosynthesis in plants: opening the door to azadirachtin production

Hannah Hodgson[a,*], Ricardo De La Peña[b,*], Michael J. Stephenson[a], Ramesha Thimmappa[a], Jason L. Vincent[c], Elizabeth S. Sattely[b,d] and Anne Osbourn[a,1].

[a] Department of Metabolic Biology, John Innes Centre, Norwich Research Park, Norwich NR4 7UH, UK; [b]Department of Chemical Engineering, Stanford University, Stanford, CA 94305, US; [c]Syngenta Ltd., Jealott's Hill International Research Centre, Bracknell, Berkshire, RG42 6EY, UK; [d]Howard Hughes Medical Institute, Stanford University, Stanford, CA 94305.

[*]These authors contributed equally to this work

[1]To whom correspondence may be addressed. Email: anne.osbourn@jic.ac.uk

**This PDF file includes:**

Supplementary text
Figs. S1 to S9
Tables S1 to S12
References for SI reference citations



www.pnas.org/cgi/doi/10.1073/pnas.1906083116

## Supplementary Information Text

## Materials and Methods

***Citrus sinensis* material.** A 12-18 month old *Citrus sinensis* var. Valencia (Sweet Orange) plant (~12 in tall) was purchased from Four Winds Growers (USA) in summer 2015. The plant was maintained outdoors in warm weather.

**RNA extraction and cDNA synthesis from *Melia azedarach and Citrus sinensis.*** *M. azedarach* and *C. sinensis* tissues were flash-frozen in liquid nitrogen and ground using a pestle and mortar. For *M. azedarach*, RNA was extracted from leaf tissues using the modified protocol for RNeasy Plant Mini Kit (Qiagen) developed for extraction from woody plants (1). For *C. sinensis*, RNA was extracted from fruit buds using Protocol A from Spectrum™ Plant Total RNA Kit (Sigma-Aldrich). DNAase treatment was performed 'on-column' using DNAse (Promega). Following the manufacturer's instructions, first-strand cDNA synthesis was performed using the GoScript™ Reverse transcription system (Promega) for *M. azedarach* samples and SuperScript™ III Reverse transcriptase (Invitrogen) for *C. sinensis* samples.

***Saccharomyces cerevisiae* heterologous recombination and transformation.** Heterologous recombination was performed in the *S. cerevisiae* strain GIL77 (*MATa/**α** gal2 hem3-6 erg7 ura3-176*) (2). Sequences were amplified by PCR using primers with homologous sequences to the ends of linearized pYES2 vector cut at restriction sites XbaI and HindIII. The 5' of the forward primer overlapped with GAL1 promoter and that of the reverse primer with the CYC1 terminator sequence. Coding sequences of oxidosqualene cyclase (OSC) candidates from *Azadirachta indica* and *C. sinensis* were synthesized by Integrated DNA Technologies (IDT) in two fragments. Fragment 1 was amplified with a pYES2-specific forward primer as described and a reverse primer with the 3' end complementary to the 5' end of fragment 2. Fragment 2 was amplified using a forward primer with the 5' end complementary to the 3' of

fragment 1 and a pYES2-specific reverse primer as described above. Coding sequences of oxidosqualene cyclase (OSC) candidates from *M. azedarach* were amplified from cDNA by PCR with pYES2-specific primers. All PCR reactions were performed using Phusion polymerase (Promega) following the manufacturer's instructions, and all primers (Table S10) were ordered from Sigma-Aldrich. The PCR fragments were co-transformed into GIL77 with linearized pYES2 vector following a standard protocol (YeastMaker™, Yeast transformation system 2, Clontech laboratories). To confirm successful recombination and correct coding sequence of PCR fragments, plasmids were extracted using Zymoprep™ Yeast Plasmid Miniprep (Zymo Research), transformed into *Escherichia coli* for propagation and extracted for sequencing. All plasmid purifications from *E. coli* were performed using QIAprep Spin Miniprep Kit (Qiagen).

**Expression of OSCs and cytochrome P450s (CYPs) in *S. cerevisiae* and triterpene extraction.** *S. cerevisiae* strains GIL77 (2) and Y21900 (*MATa/α ura3Δ0 leu2Δ0 his3Δ1 met15Δ0/MET15 LYS2/lys2Δ0 ERG7/ERG7::kanMX4*) (EuroScarf) were used for expression of candidate OSCs and CYP genes. Both strains have either partial (Y21900) or full (GIL77) loss of function of *ERG7*. All media used for GIL77 strain were supplemented with 20 µg/mL ergosterol (Fluka), 13 µg/mL hemin (Sigma-Aldrich) and 5 mg/mL Tween 80 (Sigma-Aldrich). Selection media are listed in Table S12. Strains were grown in liquid culture at 30ºC with shaking at 200 rpm. For expression of candidate genes, strains were first pre-cultured to saturation (~48 h) in SD +glucose (2% wt/vol) +[supplements]. Cells were then pelleted by centrifugation, washed in dd$H_2O$, resuspended in SD +galactose (2 % wt/vol) + [supplements] and cultured for a further 48 h before being pelleted for extraction. Cells were saponified by resuspending in 250 µl of saponification reagent (20% (wt/vol) KOH in 50% (vol/vol) EtOH) and incubating for 2 h at 65ºC. Triterpenes were then extracted in an equal volume of hexane and the hexane extracts were pooled and dried down.

**Gateway® cloning of OSCs and *M. azedarach* CYPs.** The coding sequence of candidate OSCs and CYPs were amplified by PCR with a forward primer containing 5' AttB1 site and a reverse containing 5' AttB2. Gel electrophoresis was used to confirm the sizes of PCR fragments. These were then purified using QIAquick Gel Extraction Kit or QIAquick PCR Purification Kit (Qiagen). Gateway® technology (Invitrogen) was used following the manufacturer's instructions. Briefly, purified PCR fragments were transferred into donor vector pDNR207 by performing a BP recombination reaction followed by transformation into *E. coli* (DH5α™ (ThermoFisher Scientific)). Plasmids were sequenced to check for successful recombination and correct coding sequence. Finally, an LR recombination reaction was performed to transfer the coding sequence of candidate genes from pDNR207 to the desired expression vector following the manufacturer's instructions. For expression in yeast, PYES2-DEST52 (Thermo-Fisher Scientific), pAG423GAL and pAG425GAL (Addgene) were used as expression vectors. The pEAQ-*HT*-DEST1 vector (3) (kindly provided by Lomonossoff laboratory) was used as an expression vector for *Agrobacteria tumefaciens* mediated transient expression in *Nicotiana benthamiana.*

**Gibson Assembly and cloning of *C. sinensis* CYPs.** Gene sequences for *Citrus sinensis* CYPs were amplified by PCR using Q5® High-Fidelity DNA Polymerase and gene specific primers form Integrated DNA Technologies. Gel electrophoresis was used to confirm the sizes of PCR fragments. These were then purified using QIAquick Gel Extraction Kit (Qiagen). Gibson assembly was used to insert PCR amplicons into AgeI and XhoI (New England Biolabs) linearized pEAQ-*HT*-DEST1 vector (3) (kindly provided by Lomonossoff laboratory). All CYPs were amplified with 5' pEAQ-HT vector overlaps necessary for Gibson assembly. Constructs were transformed into *E. coli* 5-alpha chemically competent cells (New England Biolabs). Plasmid DNA was isolated using the QIAprep Spin Miniprep Kit (Qiagen). Sequence confirmation was carried using Sanger DNA sequencing (Elim Biopharm).

**GC-MS analysis of triterpene extracts.** Dried samples were resuspended in 200 µL of extraction solvent and 50 µL aliquots were dried down under $N_2$ gas. Dried aliquots were then derivatized in 50µL 1-(trimethylsilyl)imidazole - pyridine mixture (Sigma-Aldrich) and heated at 65°C for 30 min, before being transferred to glass inserts in glass autosampler vials. GC-MS analysis was performed using a 7890B GC (Agilent) and an electron-impact (EI) 5977AMSD (Agilent) fitted with a Zebron ZB5-HT Inferno column (Phenomenex) following a previously described method (4). Briefly, 1 µL of sample was injected (inlet 250°C) in pulse splitless mode (pulse pressure 30 psi) with a program that involved an oven temperature at 2 min 170°C, ramp of 20 °C/min to 300°C and 11.5 min at 300 °C. Detection was carried out in scan mode (60-800 mass units), set to 7.2 after a solvent delay of 8 minutes. Data analysis was undertaken using MassHunter workstation (Agilent) software.

**General considerations for NMR.** NMR spectra were recorded in Fourier transform mode at a nominal frequency of 400 MHz for 1H NMR , and 100 MHz for 13C NMR (unless specified otherwise), using the specified deuterated solvent. Chemical shifts were recorded in ppm and referenced to the residual solvent peak or to an internal TMS standard. Multiplicities are described as, s = singlet, d = doublet, dd = doublet of doublets, dt = doublet of triplets, t = triplet, q = quartet, quint = quintet, tquin = triplet of quintets, m = multiplet, br = broad, appt = apparent; coupling constants are reported in hertz as observed and not corrected for second order effects.

**Purification of tirucalla-7,24-dien-3ß-ol (AiOSC1 product).** Two L of GIL77 cells expressing AiOSC1 (pYES2) were cultured and pelleted, yielding 15.28 g of material. Saponification was performed in 100 ml of reagent and triterpenes were extracted by addition of an equal volume of hexane in triplicate yielding 220 mg of dried crude extract. Fractionation using Isolera™ Prime (Biotage) (Table S11)

yielded 1 mg of purified tirucalla-7,24-dien-3ß-ol enabling structural confirmation by NMR.

***Azadirachta indica*** **differential gene expression analysis (DGE) and hierarchal clustering.** DGE analysis was performed using Trinity-assembled *A. indica* transcriptome (Ai1) (Table S1) as reference sequence with corresponding raw RNAseq reads from fruit, root, leaf, stem and flower tissues (5, 6). Transcript abundance estimation was performed using a script provided within the Trinity *de novo* assembler package “align and estimate abundance” (7). Briefly, raw RNAseq reads for each tissue were aligned to the transcriptome (BowTie V1.0.1 (8)) and abundance per gene was estimated (RSEM V1.3.0 (9)) using Trinity transcripts as a proxy for genes. The resultant estimated counts per gene were converted to integers and genes scoring less than one count per million in two or more tissues were excluded from the analysis. Data were normalized to account for differences in library size by using a trimmed mean of M-values (TMM) method (EdgeR V3.22.5 (10)). Due to a lack of replicates in the published dataset, a dispersion value could not be calculated and was therefore manually estimated at 0.05. A genewise negative binomial generalized linear model (EdgeR V3.22.5 (10)) was used to identify differentially expressed genes (likelihood ratio test=1 and p-value<0.05). $Log_2$-normalised read counts (DESeq2 V1.22.1(11)) were used for hierarchal clustering analysis of differentially expressed genes. Correlation matrices for tissues and genes were calculated using the Spearman (12) and Person (13) methods, respectively. Conversion of correlation matrices to distance matrices was performed based on complete linkages. The dendrogram of clustered genes was cut at 0.08422892 (max height of tree/4.85) and visualized using Heatmap3 V1.1.1 (14).

**Extraction of triterpene extracts from *N. benthamiana* leaves infiltrated with *C. sinensis* CYPs.** Leaf tissue was collected using a 1 cm DIA leaf disc cutter. Four leaf discs from the same leaf (approx. 0.04 g FW leaves) were placed inside a 2 mL safe-lock microcentrifuge and lyophilized. 500 μL of ethyl acetate was

added to each sample and these were then homogenized in a ball mill using 5 mm stainless steel beads at 25 Hz for 2 min (Retsch MM 400). Samples were placed on a shaker and incubated at room temperature for 2 hours at 300 rpm. After incubation, the samples were briefly centrifuged. 450 μL of extract were transferred to a new microcentrifuge tube and evaporated to dryness under $N_2$. The samples were reconstituted in 45 μL of methanol before LC-MS analysis.

**LCMS analysis of triterpene extracts from N. benthamiana leaves infiltrated with M. azedarach CYPs*.*** LC-MS was carried out based on a previously described method (15) using positive mode electrospray LC-MS on a Nexera/Prominence UHPLC equipped with an ion-trap ToF mass spectrometer (Shimadzu). Separation was on a 100 × 2.1 mm 100 Å 2.6 μm Kinetex EVO C18 column (Phenomenex) using 0.1% formic acid in water (A) versus methanol (B) run at 500 μL/min, 40ºC and following gradients of solvent B; 32-60% from 0-3 min, 60-65% from 7-13 min, 65-90% from 13-13.5 min, 90% from 13.5-16.5 min, 90-40% from 16.5-17 min and 40% 17-20 min. Full MS spectra were collected (m/z 200-2000) with a maximum ion accumulation time of 20 msec, and automatic sensitivity control set to a target of 70% optimal base peak intensity. The instrument also collected data-dependent MS2 (m/z 50-2000) of the most abundant precursor ions, with an isolation width of m/z 3.0, 50% collision energy and 50% collision gas, and a fixed ion accumulation time of 10 msec. Spray chamber conditions were 300ºC heat block, 250ºC curved desorbation line, 1.5 L/min nebuliser gas, and drying gas 'on'. The instrument was calibrated using sodium trifluoroacetate cluster ions according to the manufacturer's instructions. To improve separation of melianol and dihydroniloticin the above gradient was modified to 0.1% formic acid in water (A) versus methanol (B) run at 500 μL/min, 40ºC and the following gradients of solvent B; 70-95% from 0-10 min, 95% from 10-11 min, 95-70% from 11-11.1 min and 70% from 11.1-14.5 min.

**LCMS analysis of triterpene extracts from N. benthamiana leaves infiltrated with C. sinensis CYPs*.*** LC-MS was carried using mixed mode ionization (MMI:

ESI + APCI) on positive mode on an Agilent 1290 Infinity II UHPLC coupled to an Agilent 6545 Q-TOF mass spectrometer. Separation was on a 50 x 2.1 mm, 1.8 µm ZORBAX RRHD Eclipse Plus C8 column (Agilent) using 0.1% formic acid in water (A) versus methanol (B) run at 600 µL/min, 50ºC. The following gradients of solvent B were used: 3% 0 – 0.40min, 3%-97% 0.40-18.40 min, 97% 18.40-19.20 min, 97-3% 19.20-19.40 min, 3% 19.4-19.8min. Full MS spectra was collected (m/z 50 – 1700). The MMI source was set as follows: 250ºC gas temperature, 250ºC vaporizer, 2.5 L/min drying gas, 20 psi nebulizer, 1000 V VCap, 4 µA corona, and 2000 V chagrining voltage.

**Purification of dihydroniloticin (the product of AiOSC1 and MaCYP71CD2).** Using the previously described vacuum infiltration method (4, 16) 144 *N. benthamiana* plants were agroinfiltrated with equal volumes of *A. tumefaciens* strains containing pEAQ-*HT*-DEST1 expression construct for *AstHMGR, AiOSC1* and *MaCYP71CD2.* Initial extraction was performed on dried leaf material (68.64 g) following the large-scale triterpene extraction protocol previously described (16). Successive rounds of fractionation were performed using Isolera™ Prime (Biotage) as described in Table S11*.* To achieve final purification, the sample was dissolved in a minimal amount of ethanol and agitated (15 min) with activated charcoal (Sigma-Aldrich). This yielded 86 mg of dihydroniloticin, enabling structural confirmation by NMR.

**Purification of tirucalla-7,24-dien-3ß,21-diol (the product of AiOSC1 and MaCYP71BQ5).** Five L of *S. cerevisiae* Y21900 cells expressing AiOSC1 (pYES2), AtATR2 (pAG425gal) and MaCYP71BQ5 (pAG423gal) were cultured and pelleted. Saponification was carried out in 500 ml of reagent. Extraction was performed in 1.5 L of hexane, yielding 1.13 g of dried crude extract. Successive fractionation using Isolera™ Prime (Biotage) (Table S11) yielded 4 mg of tirucalla-7,24-dien-3ß,21-diol, enabling structural confirmation by NMR.

**Purification of melianol (the product of AiOSC1, MaCYP71CD and MaCYP71BQ5).** Using vacuum infiltration (4, 16) *N. benthamiana* plants (160) were agroinfiltrated with equal volumes of *A. tumefaciens* strains containing pEAQ-*HT*-DEST1 expression constructs of *AstHMGR, AiOSC1, MaCYP71CD2* and *MaCYP71BQ5.* Initial extraction was performed on dried leaf material (198.5 g) following the large-scale triterpene extraction protocol previously described (16). Successive rounds of fractionation were performed using Isolera™ Prime (Biotage) (Table S11). To achieve final purification, re-crystallisation was performed by dissolving the sample in a minimal volume of methanol (90°C), covering and allowing crystals to form at room temperature. Crystals were washed in ice-cold methanol. The re-crystallisation process was repeated, yielding ~6 mg of melianol. Structural confirmation was carried out by NMR.

**Protolimonoids**

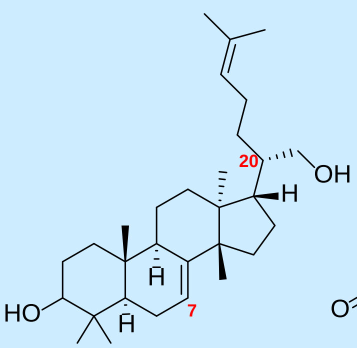

tirucalla-7,24-dien-3ß,21-diol
*Picrasma quassioides*

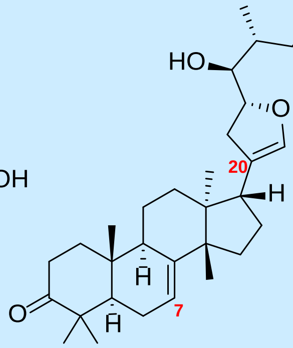

nimbocinone
*Azadirachta indica*

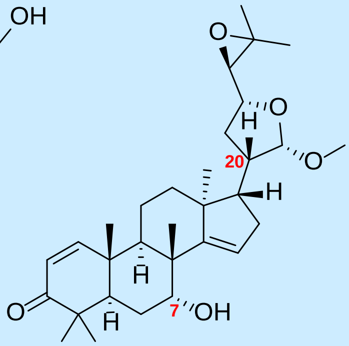

7-deacetylbruceajavanin B
*Turraea pubescens*

**Rutaceae limonoids**

*seco-A,D*-limonoid examples

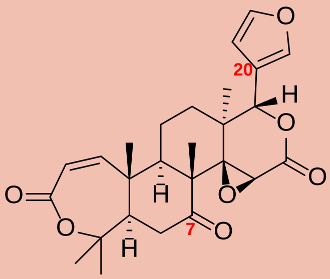

obacunone
Commonly isolated

**Meliaceae limonoids**

Ring-intact examples

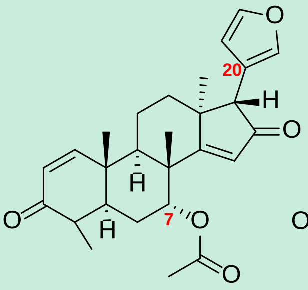

azadiradione
*Azadirachta indica*

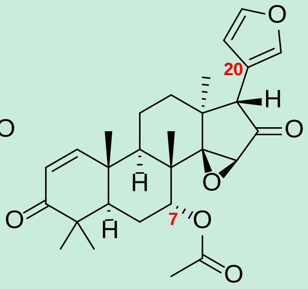

epoxyazadiradione
*Azadirachta indica*

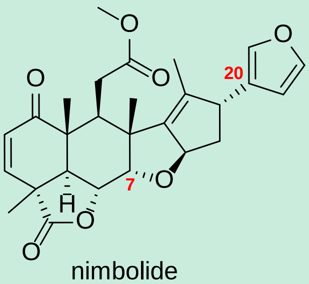

nimbolide
*Azadirachta indica*

**Fig. S1. Structures of additional protolimonoids and family-specific limonoids referred to in this work.**

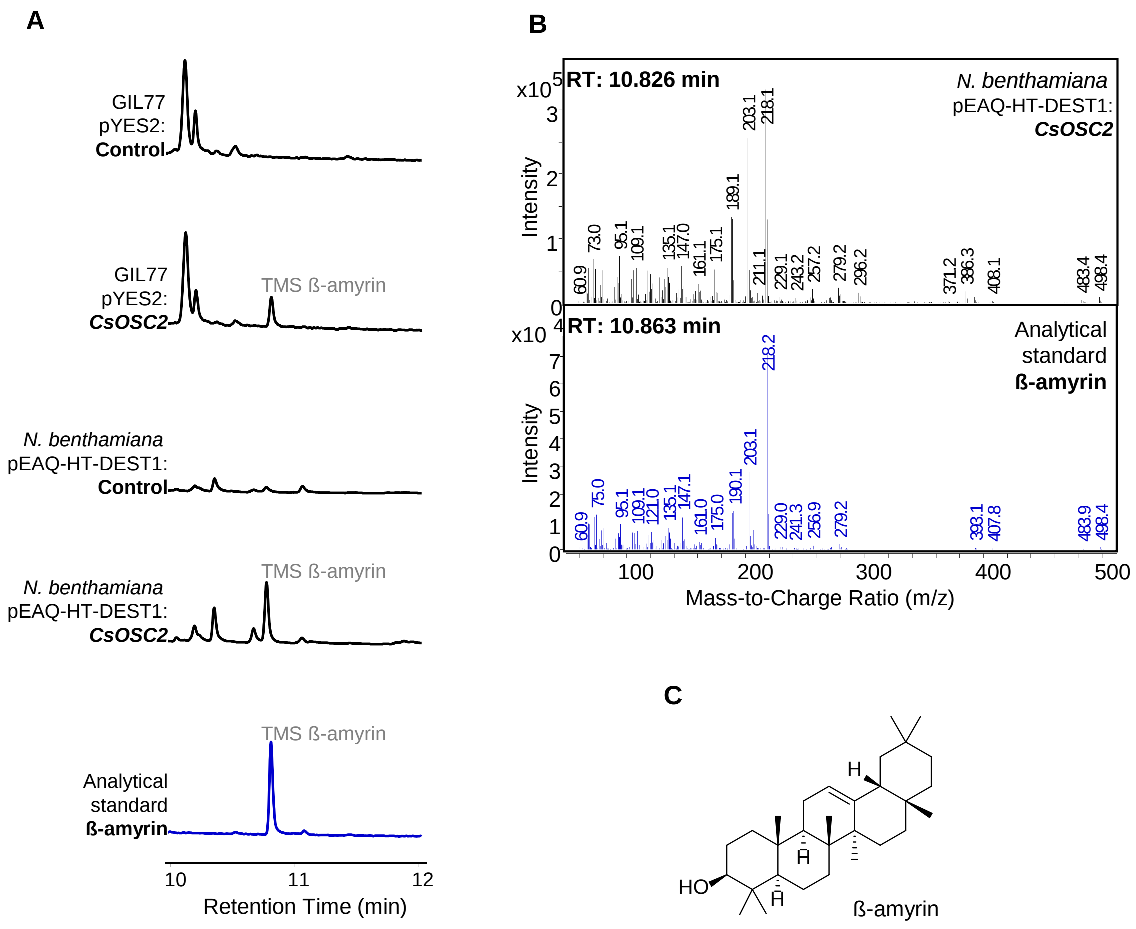


**Fig. S2. Functional characterization of CsOSC2 as a ß-amyrin synthase.**

(A) GC-MS total ion chromatograms of derivatized hexane extracts from yeast strains and ethyl acetate extracts from agroinfiltrated *N. benthamiana* leaves expressing CsOSC2 in pYES2 and pEAQ-*HT*-DEST1 vectors, respectively. Traces for the empty vector controls and for the derivatized analytical ß-amyrin standard (Sigma-Aldrich) are included. (B) GC-MS mass spectra of TMS ß-amyrin from agroinfiltrated *N. benthamiana* leaves (black) and the ß-amyrin analytical standard (blue).  (C) Structure of ß-amyrin.

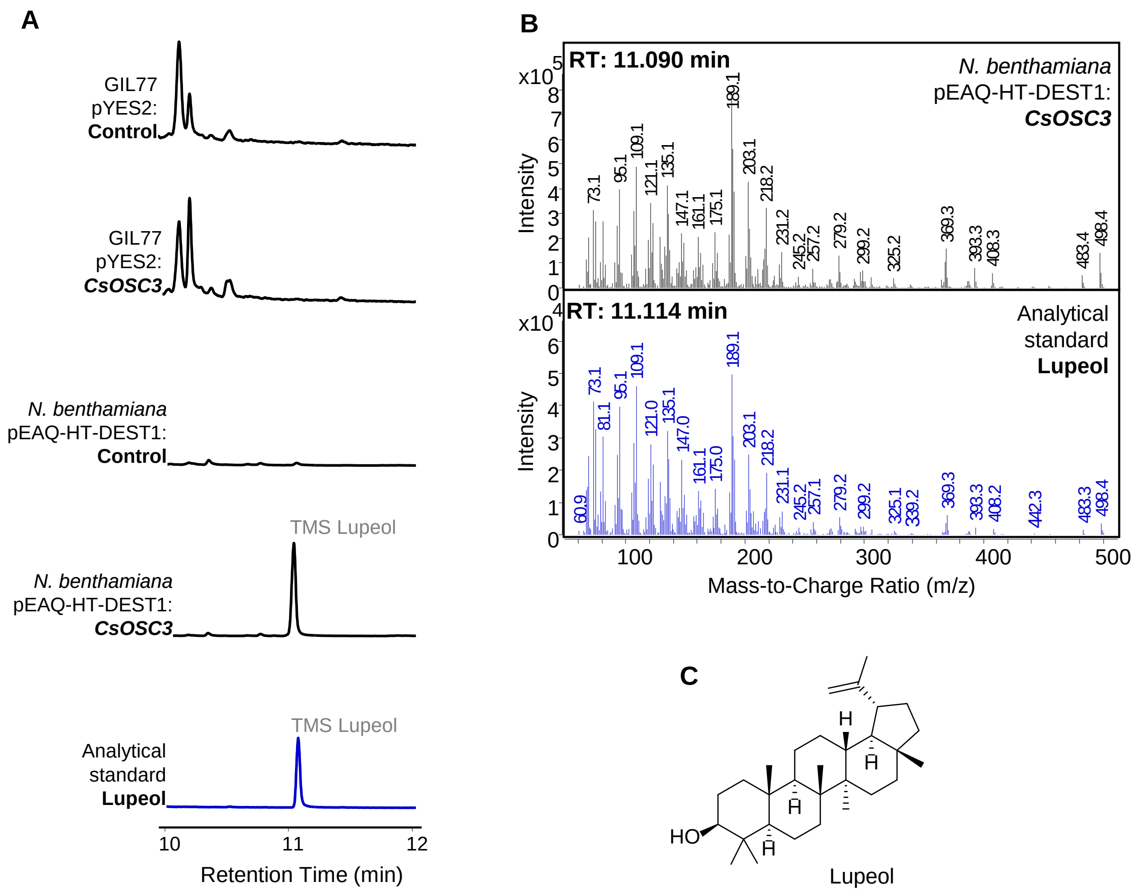


**Fig. S3. Functional characterization of CsOSC3 as a lupeol synthase.**

(A) GC-MS total ion chromatograms of derivatized hexane extracts from yeast strains and ethyl acetate extracts from agroinfiltrated *N. benthamiana* leaves expressing CsOSC3 in pYES2 and pEAQ-*HT*-DEST1 vectors, respectively. Traces for the empty vector controls and the derivatized analytical lupeol standard (Sigma-Aldrich) are included. (B) GC-MS mass spectra of TMS lupeol from agroinfiltrated *N. benthamiana* leaves (black) and analytical standard (blue). (C) Structure of lupeol.

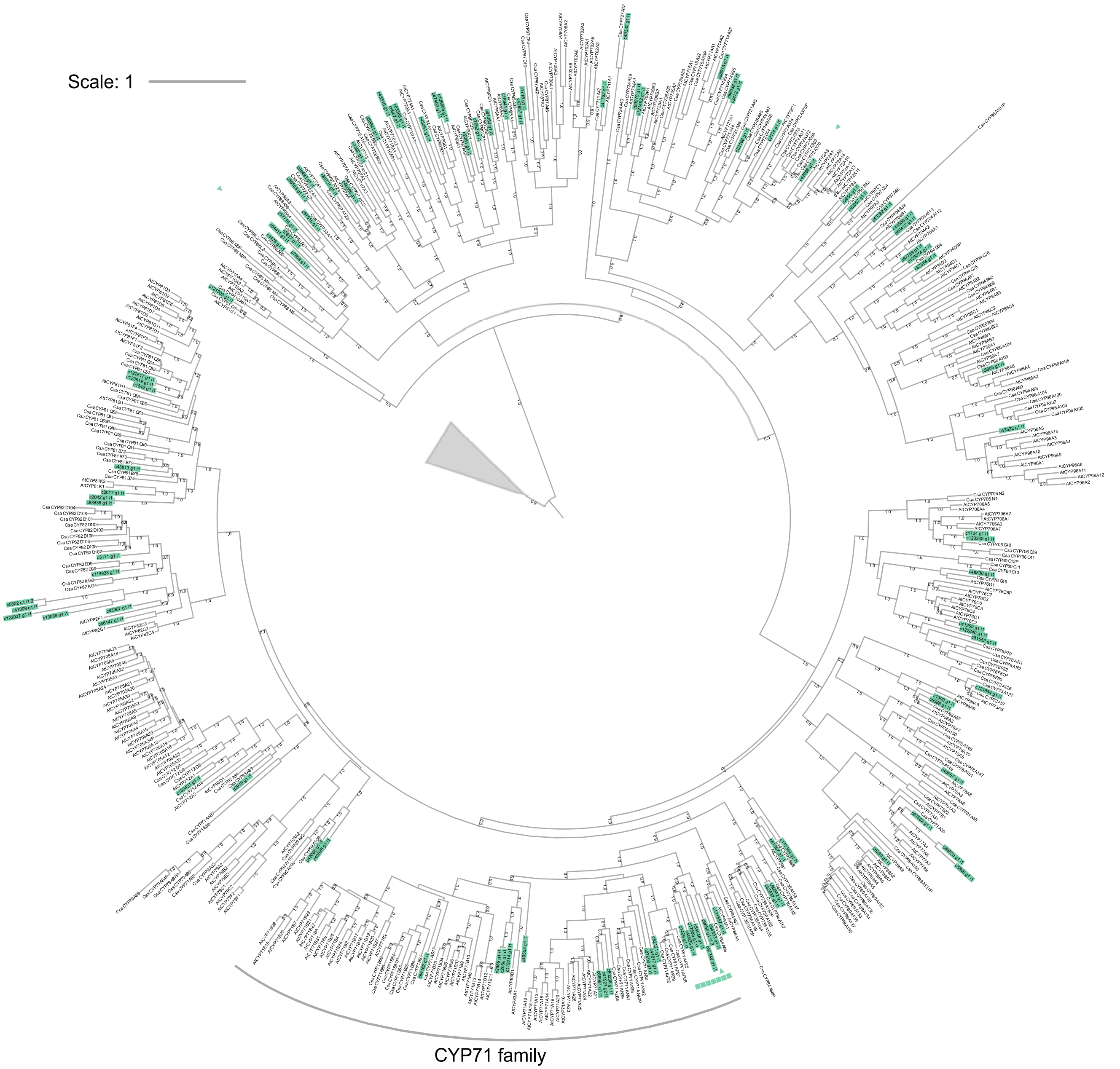


**Fig. S4 . Full version of the phylogenetic tree shown in part in Fig. 5A.**
Phylogenetic tree of candidate cytochrome P450s (CYPs) from *M. azedarach* (green) with previously identified CYPs from *Arabidopsis thaliana* (http://www.p450.kvl.dk) and C*ucumis sativus* (http://drnelson.uthsc.edu/cytochromeP450.html) (black) included. The CYP74 family is used as an outgroup (grey triangle) and the CYP71 clade is labelled based on previously described phylogeny (17). Candidate CYPs selected for cloning (Table S4) were identified by homology to A. indica candidate CYPs that were co-expressed with AiOSC1 (triangles) or occurrence in a unique CYP71 subclade lacking close homologs from *A. thaliana* or *C. sativus* (squares). The phylogenetic tree was constructed using FastTree V2.1.7 (18) and formatted with iTOL (19). Local support values from FastTree Shimodaira-Hasegawa (SH) test (between 0.6 and 1.0) are indicated at the nodes. The scale bar indicates estimated number of amino acid substitutions per site.

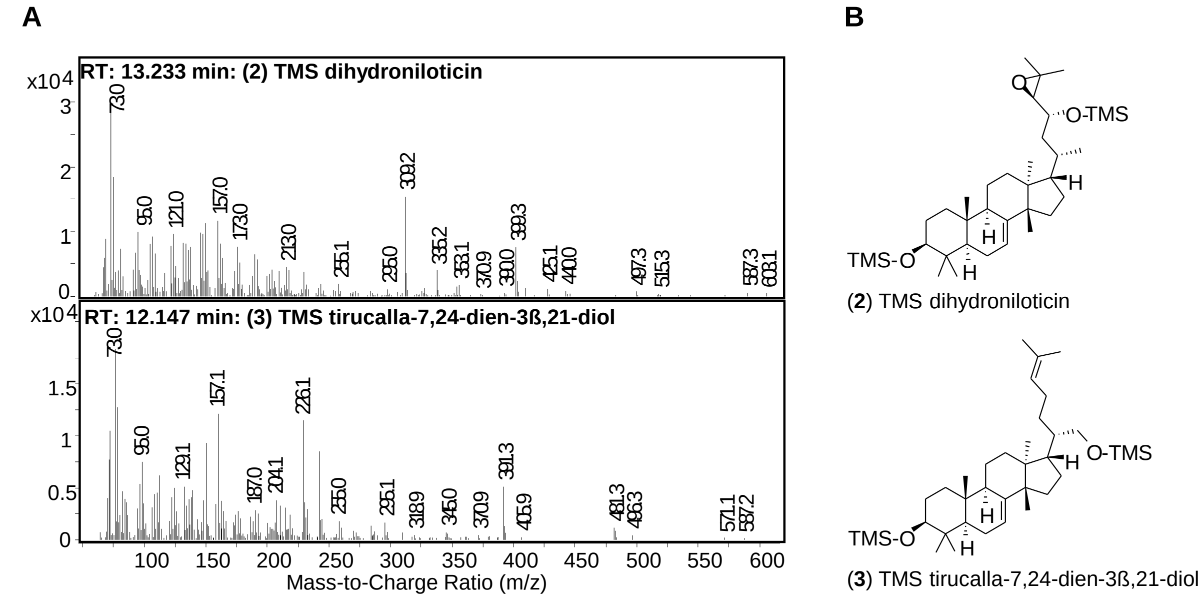


**Fig. S5. Mass spectra of TMS-dihydroniloticin (2) and TMS-tirucalla-7,24-dien-21,3ß-ol (3) generated by GC-MS.**

(A) Mass spectra corresponding to GC-MS total ion chromatograms, depicted in Fig. 5*B*, of derivatized triterpene extracts from agroinfiltrated *Nicotiana benthamiana* leaves expressing candidate genes in pEAQ-*HT*-DEST1. (B) Structures of TMS-dihydroniloticin (**2**) and TMS-tirucalla-7,24-dien-21,3ß-ol (**3**).

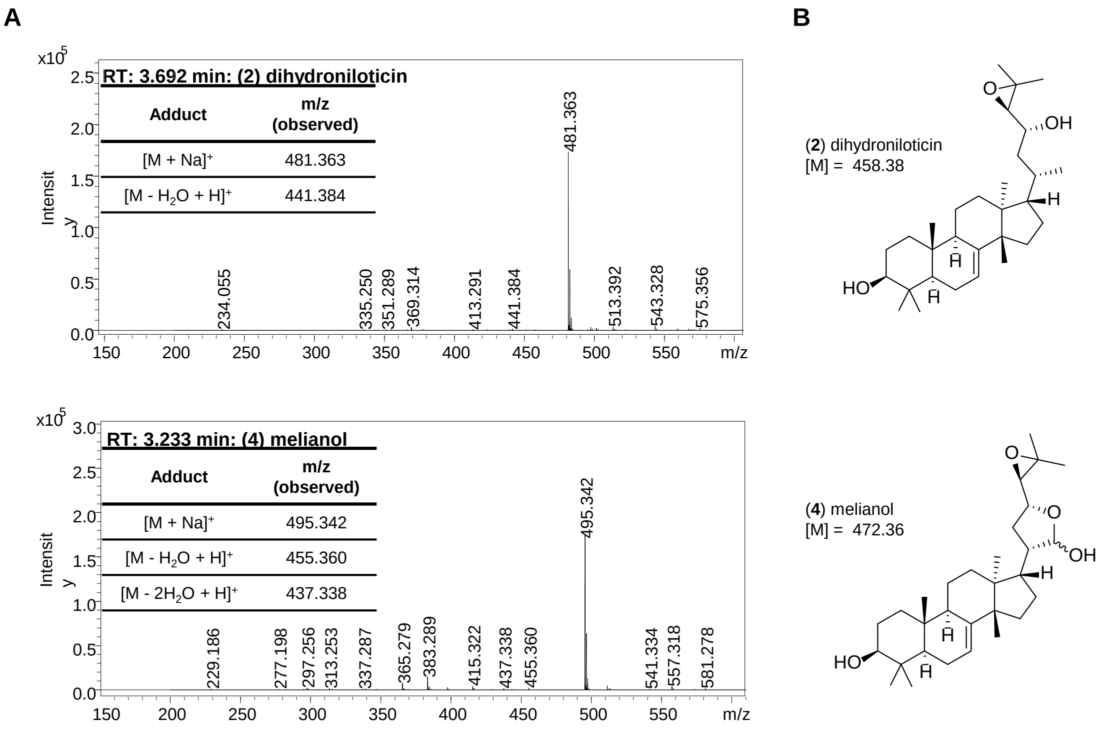


**Fig. S6. Mass spectra of dihydroniloticin (2) and melianol (4) generated by (+)-UHPLC-IT-TOF ESI.**

(A) Mass spectra corresponding to LC-MS extracted ion chromatograms, depicted in Fig. 5*C*, of triterpene extracts from agroinfiltrated *Nicotiana benthamiana* leaves expressing candidate Meliaceae genes in pEAQ-*HT*-DEST1. Observed adducts are listed. (B) Structures and exact mass of dihydroniloticin (**2**) and melianol (**4**).

**A**

**B**

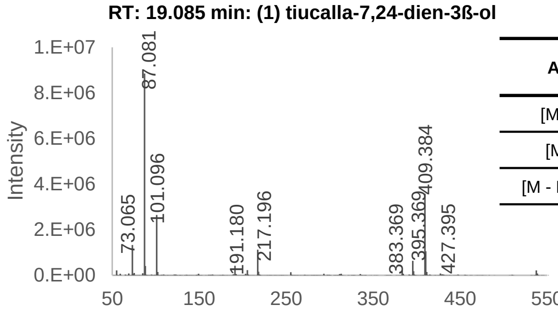


| Adduct | m/z (observed) |
|---|---|
| $[M + Na]^+$ | 449.376 |
| $[M + H]^+$ | 427.395 |
| $[M - H_2O + H]^+$ | 409.384 |

(**1**) tirucalla-7,24-dien-3ß-ol
[M] = 426.39

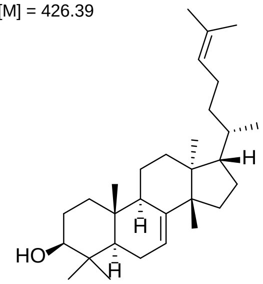


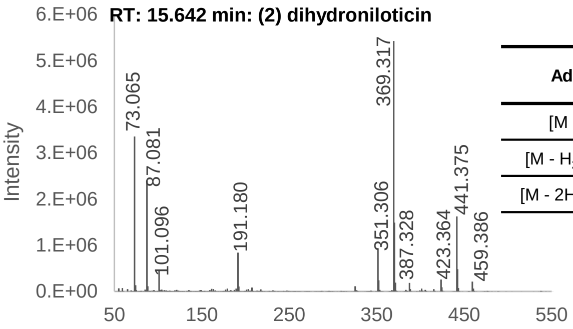


| Adduct | m/z (observed) |
|---|---|
| $[M + H]^+$ | 459.386 |
| $[M - H_2O + H]^+$ | 441.375 |
| $[M - 2H_2O + H]^+$ | 423.364 |

(**2**) dihydroniloticin
[M] = 458.38

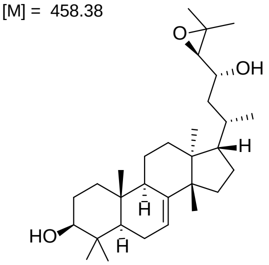


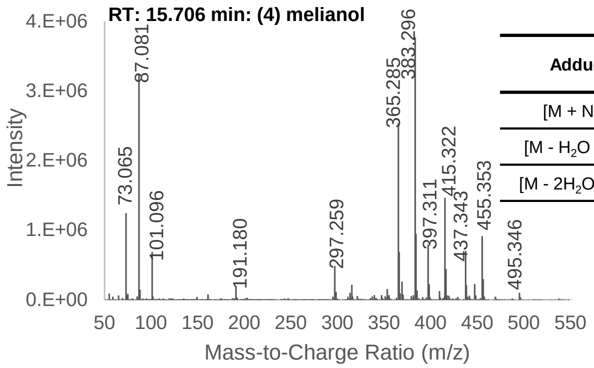


| Adduct | m/z (observed) |
|---|---|
| $[M + Na]^+$ | 495.346 |
| $[M - H_2O + H]^+$ | 455.353 |
| $[M - 2H_2O + H]^+$ | 437.343 |

(**4**) melianol
[M] = 472.36

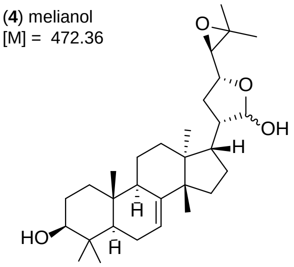


**Fig. S7. Mass spectra of tirucalla-7,24-dien-3ß-ol, dihydroniloticin (2) and melianol (4) generated by (+)-UHPLC-Q-TOF MMI.**

(A) Mass spectra corresponding to LC-MS extracted ion chromatograms, depicted in Fig. 5*D*, of triterpene extracts from agroinfiltrated *Nicotiana benthamiana* leaves expressing candidate Rutaceae genes in pEAQ-*HT*-DEST1. Observed adducts are listed. (B) Structures and exact mass of tirucalla-7,24-dien-3ß-ol (**1**), dihydroniloticin (**2**) and melianol (**4**).

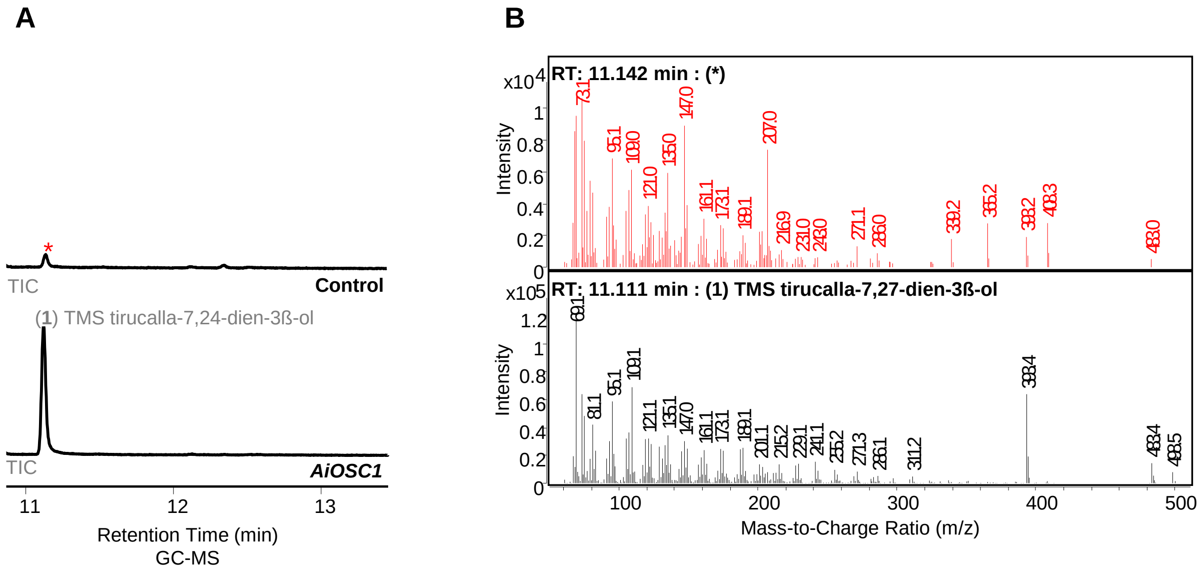


**Fig. S8. Mass spectra of co-eluting peak (*) compared to tirucalla-7,24-dien-3ß-ol (1).**

(A) GC-MS total ion chromatograms of extracts from *N. benthamiana* expressing *AiOSC1* and control. Tirucalla-7,24-dien-3ß-ol (**1**) (black) and co-eluting peak (red asterisk) are indicated. (B) Mass spectra generated by GC-MS of tirucalla-7,24-dien-3ß-ol (**1**) and co-eluting peak (red).

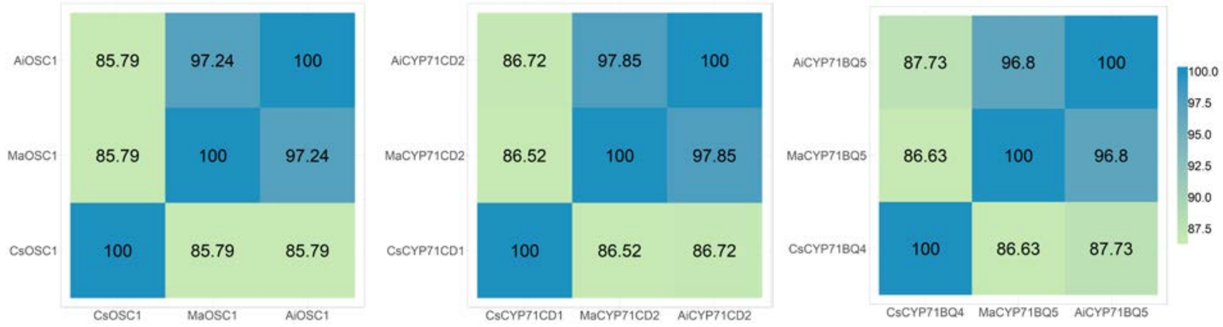


**Fig. S9. Percentage protein identities of OSC1, CYP71CD2 and CYP71BQ5 orthologs and homologs.**

Percentage protein identities calculated by Clustal Omega (20) for MaOSC1, MaCYP71CD2 and MaCYP71BQ5 and their orthologs from *Azadirachta indica* and closest homologs from *Citrus sinensis.*

**Table S1. Summary of Meliaceae transcriptomes assembled from previously generated RNAseq data.**

| Species | ID | BioSample | SRA | Tissue | Reference | Transcripts | N50 | Total bases |
|---|---|---|---|---|---|---|---|---|
| *Azadirachta indica* | Ai1 | SAMN01084665 | SRX1074135 | Fruit | 5, 6 | 73,769 | 1,171 | 53,816,418 |
| | | SAMN01084665 | SRX157673 | Root | | | | |
| | | SAMN01084665 | SRX157674 | Leaf | | | | |
| | | SAMN01084665 | SRX157675 | Stem | | | | |
| | | SAMN01084665 | SRX157676 | Flower | | | | |
| *Azadirachta indica* | Ai2 | SAMN03941262 | SRX1122777 | Leaf<br>Flower<br>Fruit | 15 | 70,275 | 1,526 | 62,521,671 |
| *Melia azedarach* | Ma1 | SAMN04510263 | SRX1597253 | Leaf | 21 | 154,767 | 1,226 | 110,216,786 |
| | | SAMN04510265 | SRX1597254 | Leaf | | | | |
| | | SAMN04510267 | SRX1597256 | Leaf | | | | |

Each transcriptome has been assigned an ID based on species and original data set: *Azadirachta indica* (Ai1 and Ai2) and *Melia azedarach* (Ma1). Details of RNAseq data used to assemble transcriptomes include: BioSample, Sequence Read Archive (SRA) identifiers and the tissue of origin. Basic statistics from Trinity *de novo* (7) generated assemblies are also included: total number of transcripts, N50 and total number of bases assembled.

**Table S2. Cloning and functional characterization of candidate OSCs.**

| Gene | Species | Dataset | Identifier | Cloning | Product | Evidence |
|---|---|---|---|---|---|---|
| *AiOSC1* (MK803262) | *Azadirachta indica* | Ai2 | c26798_g1_i1 | Synthesis and recombination into pYES2 | Tirucalla-7,24-dien-3ß-ol | Purification and NMR |
| *MaOSC1* (MK803261) | *Melia azedarach* | Ma1 | c5546_g1_i1 | Cloning and recombination pYES2 | Tirucalla-7,24-dien-3ß-ol | Purified standard |
| *CsOSC1* | *Citrus sinensis* | Cs | XP_006468116.1 | Synthesis and recombination into pYES2 | Tirucalla-7,24-dien-3ß-ol | Purified standard |
| *CsOSC2* | *Citrus sinensis* | Cs | XP_024957905.1 | Synthesis and recombination into pYES2 | β-Amyrin | Analytical standard |
| *CsOSC3* | *Citrus sinensis* | Cs | XP_015382484.1 | Synthesis and recombination into pYES2 | Lupeol | Analytical standard |
| *AtLUP5* | *Arabidopsis thaliana* | TAIR | AT1G66960.1 | Purchased and recombination into pYES2 | Tirucalla-7,24-dien-3ß-ol | Previous characterisa tion (22) |

Details of OSCs characterized in this study including assigned gene name and species of origin. Datasets used for identification were assembled transcriptomes (Ai1, Ai2, Ma1 (Table S1)) and *Citrus sinensis* protein annotation (GCF_000317415.1 (Cs)). GenBank accession numbers for *M. azedarach* and *A. indica* sequences are also provided. Cloning strategy employed, product identified in yeast strain or *N. benthamiana* and method of identification are summarized. Evidence of product identification is provided (Fig. 2, S2-S3, Table S3).

**Table S3. $^{13}$C & $^{1}$H δ assignments for tirucalla-7,24-dien-3β-ol.**

**Carbon numbering scheme and selected COSY and HMBC**

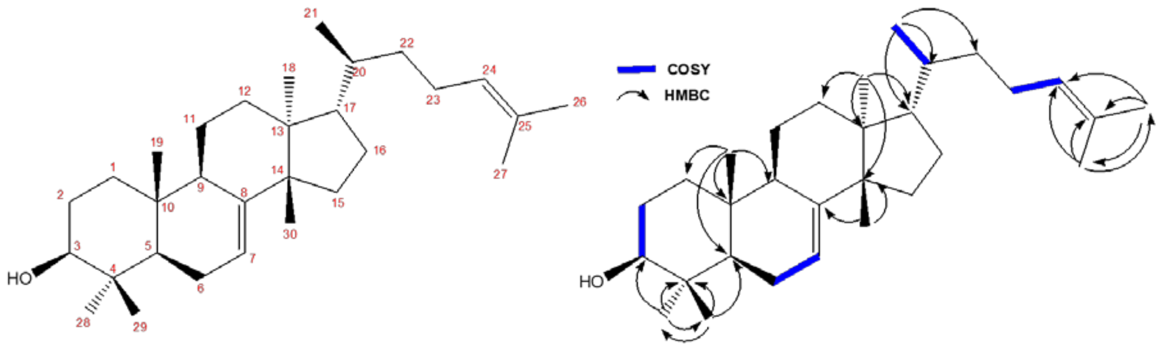


| Carbon # | $^{13}$C δ | $^{1}$H δ | Carbon # | $^{13}$C δ | $^{1}$H δ |
|---|---|---|---|---|---|
| **8** | 145.90 | / | **15** | 34.03 | 1.45 (2H, m) |
| **25** | 130.94 | / | **12** | 33.79 | 1.79 (1H, m)<br>1.61 (1H, m) |
| **24** | 125.22 | 5.01 (1H, appt tquin, *J*=7.1, 1.4) | **16** | 28.22 | 1.93 (1H, m)<br>1.27 (1H, m) |
| **7** | 117.80 | 5.25 (1H, m) | **2** | 27.70 | 1.64 (2H, m) |
| **3** | 79.27 | 3.25 (1H, dd, *J*=11.2, 4.3) | **28** | 27.61 | 0.97 (3H, s) |
| **17** | 52.95 | 1.47 (1H, m) | **30** | 27.27 | 0.97 (3H, s) |
| **14** | 51.15 | / | **27** | 25.73 | 1.68 (3H, s) |
| **5** | 50.63 | 1.32 (1H, m) | **23** | 25.02 | 2.04 (1H, m)<br>1.86 (1H, m) |
| **9** | 48.95 | 2.20 (1H, m) | **6** | 23.94 | 2.14 (1H, m)<br>1.96 (1H, m) |
| **13** | 43.52 | / | **18** | 21.91 | 0.81 (3H, s) |
| **4** | 38.96 | / | **21** | 18.33 | 0.88 (3H, d, *J*=6.4) |
| **1** | 37.21 | 1.68 (1H, m)<br>1.14 (1H, m) | **11** | 18.13 | 1.51 (2H, m) |
| **22** | 36.19 | 1.46 (1H, m)<br>1.03 (1H, m) | **26** | 17.64 | 1.60 (3H, s) |
| **20** | 35.97 | 1.36 (1H, m) | **29** | 14.73 | 0.86 (3H, s) |
| **10** | 34.95 | / | **19** | 13.12 | 0.75 (3H, s) |

NMR spectra were recorded using $CDCl_3$ and referenced to TMS. Coupling constants are reported as observed and not corrected for second order effects. Assignments were made via a combination of 1H, 13C, DEPT-edited HSQC, HMBC and 2D NOESY experiments. Where signals overlap 1H δ is reported as the centre of the respective HSQC crosspeak. Assignments were consistent with previous literature assignments for tirucalla-7,24-dien-3ß-ol (23).

**Table S4. Candidate CYPs from *M. azedarach*, orthologs from *A. indica* and closest *C. sinensis* homologs.**

| ***M. azedarach*** **(Ma1)** | ***A. indica*** **(Ai1 & Ai2)** | ***C. sinensis*** **(Cs)** | **Evidence** | **Cloning from *M. azedarach*** |
|---|---|---|---|---|
| ***MaCYP72A720*** **(MK803263)** Ma1:c42086_g1_i1 | ***AiCYP72A721**** **(MK803273)** Ai1:c10232_g3_i1**, Ai2:c60329_g1_i1**, Ai2:c45683_g1_i1** | | *A. indica* DGE | 2 SNPs: 1 amino acid change (position 1251: serine replaces proline) |
| ***MaCYP88A108*** **(MK803265)** Ma1:c5845_g1_i1, Ma1:c4476_g1_i1 | ***AiCYP88A108**** **(MK803277)** Ai1:c72218_g1_i1**, Ai2:c44421_g1_i1** | ***CsCYP88A51*** **(1 indel)** XP006485427.1 | *A. indica* DGE | 0 SNPs |
| ***MaCYP71BQ5*** **(MK803264)** Ma1:c121026_g1_i1, Ma1:c9658_g1_i1 | ***AiCYP71BQ5**** **(MK803272)** Ai1:c1285_g1_i1*, Ai2:c23690_g1_i1*, Ai2:c42255_g1_i1* | ***CsCYP71BQ4*** XP006469495.1 | *A. indica* DGE CYP71 subclade | 0 SNPs |
| ***MaCYP71CD2*** **(MK803271)** Ma1:c87349_g1_i1, Ma1:c123757_g1_i1 | ***AiCYP71CD2*** **(MK803275)** Ai1:c49952_g1_i1, Ai1:c62263_g1_i1, Ai2:c16061_g1_i1** | ***CsCYP71CD1*** XP006467299.1 | CYP71 subclade | 0 SNPs |
| ***MaCYP71BE124*** **(MK803267)** Ma1:c17683_g1_i1 | ***AiCYP71BE127*** **(MK803276)** Ai1:c62481_g1_i1 | ***CsCYP71BE38*** XP006475119.1 | CYP71 subclade | 1 SNPs: 0 amino acid change |

**Table S4 (continued). Candidate CYPs from *M. azedarach,* orthologs from *A. indica* and closest *C. sinensis* homologs*.***

| | | | | |
|---|---|---|---|---|
| ***MaCYP71D557* (MK803270)** Ma1:c54548_g1_i1 | | ***CsCYP71D416*** XP015382368.2 | CYP71 subclade | 0 SNPs |
| ***MaCYP71BQ6* (MK803269)** Ma1:c54333_g1_i1, Ma1:c127686_g1_i1 | | | CYP71 subclade | 0 SNPs |
| ***MaCYP71BE123* (MK803266)** Ma1:c1469_g1_i1 | | | CYP71 subclade | Unable to amplify |
| ***MaCYP71BE125* (MK803268)** Ma1:c40628_g1_i1 | ***AiCYP71BE126** (MK803274)** Ai1:c14914_g1_i1* Ai2:c39917_g1_i1* Ai2:c59390_g1_i1* | | CYP71 subclade | Unable to amplify |

**Table S4 (continued). Candidate CYPs from *M. azedarach,* orthologs from *A. indica* and closest *C. sinensis* homologs*.***

Candidate CYPs from *M. azedarach* transcriptome (Ma1) are listed with their orthologs or closet homologs from *A. indica* transcriptomes (Ai1, Ai2) and *C. sinensis* protein annotation (GCF_000317415.1 (Cs)). For each candidate, the CYP nomenclature is listed (black) along with identifiers in datasets (grey). GenBank accession numbers for *M. azedarach* and *A. indica* sequences are also provided. CYPs and fragments from *A. indica* and *M. azedarach* were named and assigned to clans by the Cytochrome P450 Nomenclature Committee following established convention (24). CYPs from *C. sinensis* had previously been identified from an alternative *C. sinensis* genome (25) and later assigned names by the Cytochrome P450 Nomenclature Committee (24). Instances where homologs were not identifiable in a dataset (grey box), were not full-length candidates (*), or were under 300 amino acids and therefore considered a fragment (**) are indicated. Candidate selection was based on differential gene expression analysis performed on an *A. indica* dataset (*A. indica* DGE) (5, 6) or occurrence in the unique CYP71 subclade (CYP71). Phylogeny of the CYP candidates is presented in Fig. 5*A*, with the exception of MaCYP72A720 and MaCYP88A108 which are phylogenetically distinct from the CYP71 family and therefore depicted in the full phylogenetic tree (Fig. S4). Candidates from *M. azedarach* (Ma1) were cloned and details of amplification and number of single nucleotide polymorphisms (SNPs) are given.

**Table S5. $^{13}$C & $^{1}$H δ assignments for tirucalla-7-ene-24,25-epoxy-3β,23-diol (dihydroniloctin).**

**Carbon numbering scheme and selected COSY and HMBC**

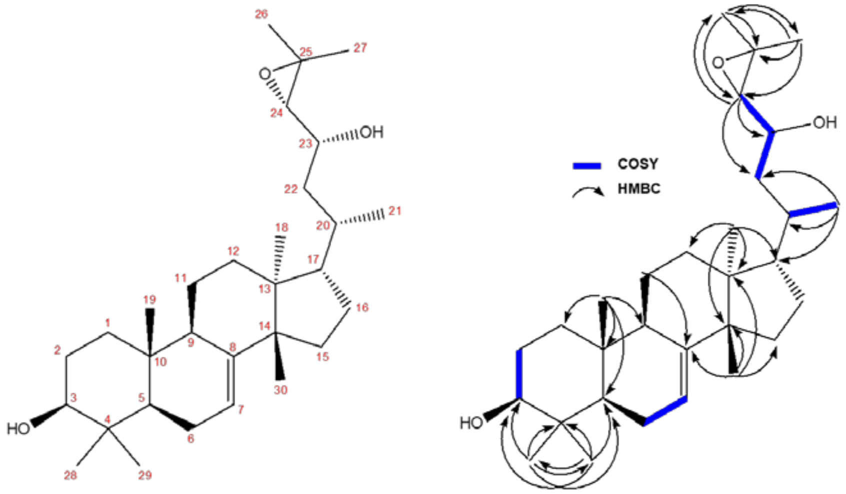


| Carbon # | $^{13}$C δ | $^{1}$H δ | Carbon # | $^{13}$C δ | $^{1}$H δ |
|---|---|---|---|---|---|
| **8** | 145.59 | / | **15** | 34.00 | 1.49 (2H, m) |
| **7** | 118.07 | 5.26 (1H, m) | **12** | 33.75 | 1.81 (1H, m)<br>1.61 (1H, m) |
| **3** | 79.25 | 3.24 (1H, dd, *J*=11.1, 4.2) | **20** | 33.60 | 1.40 (1H, m) |
| **23** | 69.32 | 3.57 (1H, td, *J*=8.2, 5.2) | **16** | 28.78 | 2.04 (1H, m)<br>1.22 (1H, m) |
| **24** | 68.45 | 2.66 (1H, d, *J*=8.2) | **2** | 27.70 | 1.65 (1H, m) |
| **25** | 60.25 | / | **28** | 27.62 | 0.97 (3H, s) |
| **17** | 53.28 | 1.56 (1H, m) | **30** | 27.24 | 0.99 (3H, s) |
| **14** | 51.21 | / | **26** | 24.88 | 1.33 (3H, s) |
| **5** | 50.65 | 1.32 (1H, m) | **6** | 23.95 | 2.15 (1H, m)<br>1.97 (1H, m) |
| **9** | 48.95 | 2.19 (1H, m) | **18** | 21.72 | 0.82 (3H, s) |
| **13** | 43.61 | / | **21** | 19.94 | 0.96 (3H, d, *J*=6.2) |
| **22** | 40.76 | 1.66 (1H, m)<br>1.41 (1H, m) | **27** | 19.82 | 1.32 (3H, s) |
| **4** | 38.98 | / | **11** | 18.10 | 1.53 (2H, m) |
| **1** | 37.22 | 1.69 (1H, m)<br>1.14 (1H, m) | **29** | 14.73 | 0.86 (3H, s) |
| **10** | 34.96 | / | **19** | 13.12 | 0.75 (3H, s) |

NMR spectra were recorded using $CDCl_3$ and referenced to TMS. Coupling constants are reported as observed and not corrected for second order effects. Assignments were made via a combination of $^{1}$H, $^{13}$C, DEPT-edited HSQC, HMBC and 2D NOESY experiments. Where signals overlap $^{1}$H δ is reported as the centre of the respective HSQC crosspeak. Assignments were consistent with previous literature assignments for dihydroniloticin (26).

**Table S6. $^{13}$C & $^{1}$H δ assignments for tirucalla-7,24-dien-3β,21-diol.**

**Carbon numbering scheme and selected COSY and HMBC**

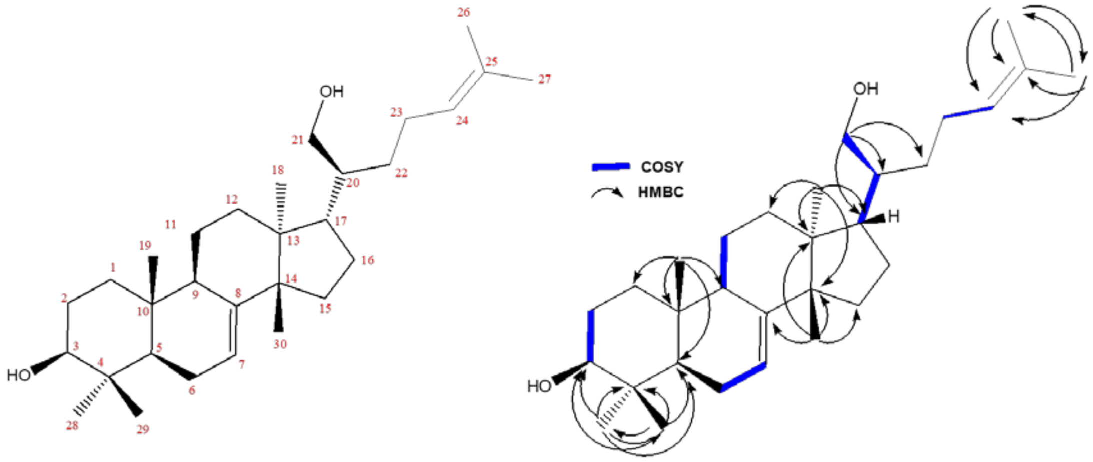


| Carbon # | $^{13}$C δ | $^{1}$H δ | Carbon # | $^{13}$C δ | $^{1}$H δ |
|---|---|---|---|---|---|
| **8** | 145.61 | / | **15** | 33.91 | 1.49 (2H, m) |
| **25** | 131.48 | / | **12** | 32.83 | 1.87 (1H, m)<br>1.61 (1H, m) |
| **24** | 124.86 | 5.12 (1H, tq, *J*=7.1, 1.3) | **22** | 29.47 | 1.44 (2H, m) |
| **7** | 118.07 | 5.27 (1H, m) | **16** | 27.81 | 1.33 (2H, m) |
| **3** | 79.24 | 3.24 (1H, dd, *J*= 11.2, 4.3) | **2** | 27.68 | 1.95 (1H, m)<br>1.65 (1H, m) |
| **21** | 62.50 | 3.73 (1H, dd, *J*=11.0, 2.9)<br>3.60 (1H, *J*=11.0, 4.9) | **29** | 27.61 | 0.97 (3H, s) |
| **14** | 51.23 | / | **30** | 27.35 | 0.99 (3H, s) |
| **5** | 50.62 | 1.31 (1H, m) | **26** | 25.72 | 1.69 (3H, s) |
| **9** | 48.84 | 2.20 (1H, m) | **23** | 24.87 | 2.06 (1H, m)<br>1.95 (1H, m) |
| **17** | 47.04 | 1.82 (1H, m) | **6** | 23.95 | 2.15 (1H, m )<br>1.97 (1H, m) |
| **13** | 43.35 | / | **18** | 22.14 | 0.83 (3H, s) |
| **20** | 42.55 | 1.46 (1H, m) | **11** | 18.03 | 1.53 (2H, m) |
| **4** | 38.96 | / | **27** | 17.73 | 1.62 (3H, s) |
| **1** | 37.19 | 1.68 (1H, m)<br>1.14 (1H, td, *J*=12.5, 4.3) | **28** | 14.72 | 0.86 (3H, s) |
| **10** | 34.95 | / | **19** | 13.11 | 0.75 (3H, s) |

NMR spectra were recorded using $CDCl_3$ and referenced to TMS. Coupling constants are reported as observed and not corrected for second order effects. Assignments were made via a combination of $^{1}$H, $^{13}$C, DEPT-edited HSQC, HMBC and 2D NOESY experiments. Where signals overlap $^{1}$H δ is reported as the centre of the respective HSQC crosspeak.

**Table S7. 13C δ comparison to the literature for tirucalla-7-ene-23,21,24,25-diepoxy-3β,21-diol (melianol) C21 epimeric mixture.**

**Carbon numbering scheme**

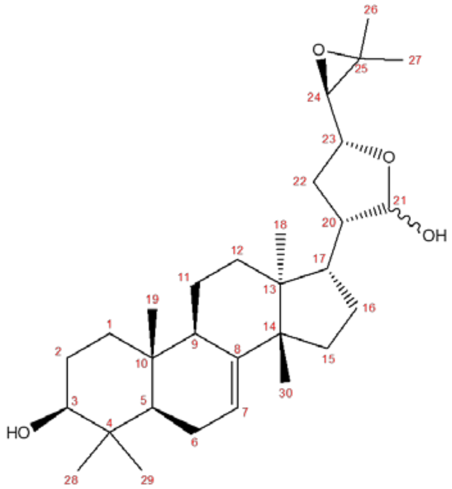


| Carbon # | Literature $^{13}$C δ (100 MHz) | | This work $^{13}$C δ (150 MHz) | | Carbon # | Literature $^{13}$C δ (100 MHz) | | This work 13C δ (150 MHz) | |
|---|---|---|---|---|---|---|---|---|---|
| **9** | 145.71 | 145.53 | 145.59 | 145.42 | **10** | 35.11 | | 35.05 | |
| **7** | 118.28 | 118.17 | 118.28 | 118.18 | **15** | 34.32 | | 34.25 | |
| **21** | 101.83 | 97.80 | 101.86 | 97.86 | **20** | 33.90 | 31.90 | 33.83 | 31.87 |
| **3** | 79.24 | | 79.25 | 79.21 | **22** | 31.61 | 31.49 | 31.52 | 31.47 |
| **23** | 78.50 | 77.02 | 78.53 | 77.12 | **2** | 27.70 | | 27.70 | |
| **24** | 67.85 | 65.49 | 67.76 | 65.34 | **28** | 27.55 | | 27.48 | 27.61 |
| **25** | 57.97 | 57.24 | 58.03 | 57.25 | **16** | 27.40 | 27.17 | 27.37 | 27.12 |
| **5** | 50.85 | 50.82 | 50.78 | 50.75 | **26** | 25.05 | 24.99 | 25.05 | 24.95 |
| **14** | 50.79 | 50.52 | 50.68 | 50.44 | **19** | 24.06 | | 23.99 | 23.98 |
| **9** | 49.67 | 48.88 | 49.71 | 48.83 | **6** | 23.27 | | 23.23 | |
| **17** | 47.17 | 45.24 | 47.15 | 45.25 | **30** | 22.64 | | 22.60 | 22.71 |
| **13** | 43.87 | 43.69 | 43.82 | 43.63 | **27** | 19.51 | 19.30 | 19.46 | 19.22 |
| **4** | 39.03 | | 38.99 | | **11** | 17.64 | | 17.56 | 17.55 |
| **1** | 37.28 | | 37.17 | 37.15 | **29** | 14.80 | | 14.72 | |
| **12** | 35.23 | | 35.24 | | **18** | 13.15 | | 13.08 | |

NMR spectra were recorded in Fourier transform mode at a nominal frequency of 150 MHz using $CDCl_3$ and referenced to TMS. Assignments were consistent with previous literature assignments for melianol (27).

**Table S8. Summary of previously isolated protolimonoids (Fig. 5*E*) and close derivatives.**

| Protolimonoids | Family | Subfamily | Species | Tissue | Reference |
|---|---|---|---|---|---|
| 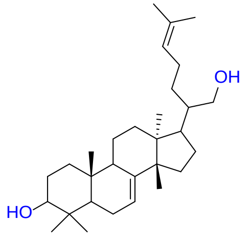<br>tirucalla-7,24-dien-21,3ß-diol | Simaroubaceae | Simarouboideae | *Picrasma quassioides* | Stem | 28 |
| 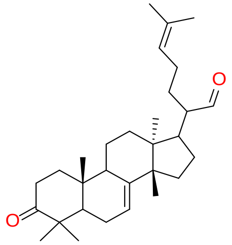<br>3-oxotirucalla-7,24-dien-21-al | Meliaceae | Melioideae | *Owenia cepiodora* | Bark | 29 |
| | Rutaceae | Aurantioideae | *Paramignya griffithii* | Stem | 30 |
| | Simaroubaceae | Simarouboideae | *Simaba cuneata* | Stem | 31 |
| | | | *Simarouba amara* | Bark | 32 |
| 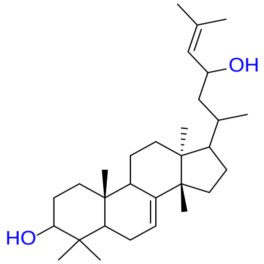<br>tirucalla-7,24-dien-23,3ß-diol | Meliaceae | Melioideae | *Dysoxylum hainanense* | Bark | 33 |
| | | | *Dysoxylum variabile* | Bark | 34 |
| | Rutaceae | Aurantioideae | *Paramignya monophylla* | Fruits | 35 |
| | Simaroubaceae | Simarouboideae | *Picrasma quassioides* | Stem | 28 |

**Table S8 (continued). Summary of previously isolated protolimonoids (Fig. *5E*) and close derivatives.**

| | | | | | |
|---|---|---|---|---|---|
| 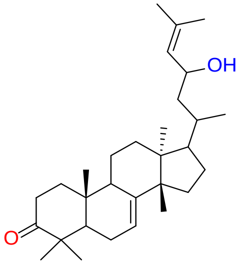<br>3-oxotirucalla-7,24-dien-23-ol | Meliaceae | Melioideae | *Dysoxylum beddomei* | Leaf | 36 |
| | | | *Dysoxylum densiflorum* | Stem | 37 |
| | Rutaceae | Aurantioideae | *Paramignya monophylla* | Fruit | 35 |
| | Simaroubaceae | Simarouboideae | *Picrasma quassioides* | Stem | 28 |
| | | | *Simarouba amara* | Bark | 38 |
| 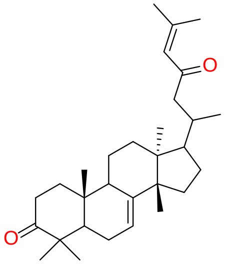<br>tirucalla-7,24-dien-3,23-dione | Meliaceae | Melioideae | *Dysoxylum macranthum* | Bark | 39 |
| 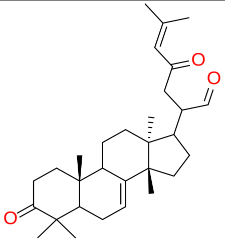<br>3,23-dioxotirucalla<br>-7,24-dien-21-al | Meliaceae | Cedreliodeae | *Entandrophragma angolense* | Leaf | 40 |

**Table S8 (continued). Summary of previously isolated protolimonoids (Fig. 5E) and close derivatives.**

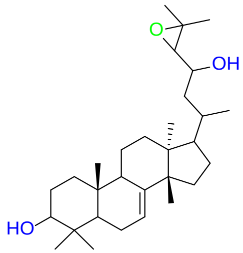

dihydroniloticin

| | | | | | |
|---|---|---|---|---|---|
| dihydroniloticin | Meliaceae | Melioideae | *Azadirachta indica* | Leaf & stem | 41 |
| | | | *Dysoxylum hainanense* | Bark | 33 |
| | | | *Dysoxylum mollissimum* | Leaf | 42 |
| | | | *Dysoxylum variabile* | Bark | 34 |
| | | | *Trichilia connaroides* | Fruit | 43 |
| | | | *Trichilia hirta* | Fruit | 44 |
| | | | *Trichilia quadrijuga* | Leaf & stem | 45 |
| | | | *Trichilia reticulata* | Leaf & stem | 46 |
| | | | *Trichilia schomburgkii* | Leaf | 47, 48 |
| | | | *Walsura robusta* | Leaf | 49 |
| | | Cedreliodeae | *Toona ciliata* | Leaf | 50 |
| | Rutaceae | Toddalioideae | *Phellodendron amurense* | Fruit | 51 |
| | | | *Phellodendron chinense* | Fruit | 26 |
| | Simaroubaceae | Simarouboideae | *Eurycoma longifolia* | Stem | 52 |
| | | | *Picrasma quassioides* | Stem | 28 |
| | | | *Simaba polyphylla* | Stem | 53 |

**Table S8 (continued). Summary of previously isolated protolimonoids (Fig. 5*E*) and close derivatives.**

| | | | | | |
|---|---|---|---|---|---|
| 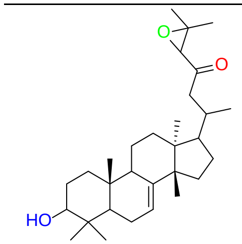 23-oxodihydroniloticin | Meliaceae | Melioideae | *Azadirachta indica* | Leaf & stem | 41 |
| 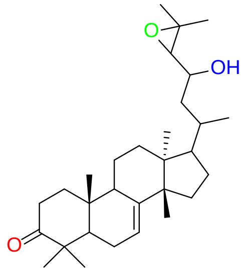 niloticin | Meliaceae | Melioideae | *Aglaia cucullata* | Stem | 54 |
| | | | *Aglaia leucophylla* | Bark | 55 |
| | | | *Dysoxylum beddomei* | Leaf | 36 |
| | | | *Dysoxylum variabile* | Bark | 34 |
| | | | *Trichilia hirta* | Fruit | 44 |
| | | | *Trichilia quadrijuga* | Leaf & stem | 45 |
| | | | *Trichilia schomburgkii* | Leaf | 48, 47 |
| | | | *Turraea nilotica* | Root | 56 |
| | | | *Walsura robusta* | Leaf | 49 |
| | | Cedreliodeae | *Toona ciliata* | Bark | 57 |

**Table S8 (continued). Summary of previously isolated protolimonoids (Fig. 5*E*) and close derivatives.**

| | | | | | |
|---|---|---|---|---|---|
| 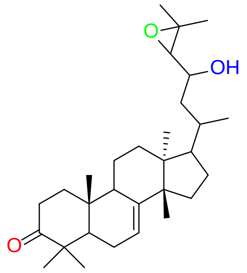<br>niloticin (continued) | Rutaceae | Rutoideae | *Boronia alata* | Leaf & stem | 58 |
| | | | *Boronia inornata* | All | 59 |
| | | Aurantioideae | *Limonia acidissima* | Leaf | 60 |
| | | | *Luvunga sarmentosa* | Leaf | 61 |
| | | Toddalioideae | *Phellodendron amurense* | Fruit | 51 |
| | | | *Phellodendron chinense* | Fruit | 26 |
| | | | *Vepris uguenensis* | | 62 |
| | Simaroubaceae | Simarouboideae | *Ailanthus altissima* | Bark | 63 |
| | | | *Castela polyandra* | Stem | 64 |
| | | | *Eurycoma longifolia* | | 52 |
| | | | *Picrasma quassioides* | Stem | 28 |
| | | | *Simarouba amara* | Bark | 38 |
| | | | *Simaba polyphylla* | Stem | 53 |
| 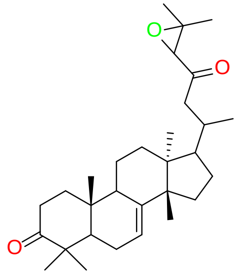<br>24,25-epoxytirucalla-7-ene -3,23-dione | Meliaceae | Melioideae | *Aphanamixis polystachya* | Bark | 65 |
| | | | *Dysoxylum laxiracemosum* | Bark | 66 |
| | | | *Dysoxylum lenticellatum* | Leaf & stem | 67 |

**Table S8 (continued). Summary of previously isolated protolimonoids (Fig. 5*E*) and close derivatives.**

| | | | | | |
|---|---|---|---|---|---|
| 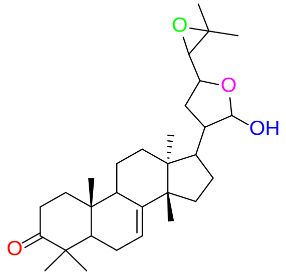 melianone | Meliaceae | Melioideae | *Dysoxylum beddomei* | Leaf | 36 |
| | | | *Guarea convergens* | Leaf | 68 |
| | | | *Guarea grandiflora* | Seed | 69 |
| | | | *Guarea kunthiana* | Fruit | 70 |
| | | | *Melia azedarach* | Fruit | 71, 72 |
| | | | *Melia toosendan* | Fruit | 27 |
| | | | *Trichilia connaroides* | Fruit | 43 |
| | | | *Trichilia hirta* | Fruit | 44 |
| | | | *Trichilia reticulata* | Leaf & stem | 46 |
| | | | *Quivisia papinae* | Seed | 73 |
| | | Cedreliodeae | *Chukrasia tabularis* | | 74 |
| | | | *Swietenia mahagoni* | | 75 |
| | Rutaceae | Aurantioideae | *Luvunga sarmentosa* | Leaf | 61 |
| | | Toddalioideae | *Phellodendron chinense* | Fruit | 76 |
| | | | *Raulinoa echinata* | Stem | 77 |
| | Simaroubaceae | Simarouboideae | *Eurycoma longifolia* | | 52 |
| | | | *Picrasma quassioides* | Stem | 28 |
| | | | *Simarouba amara* | Root | 32 |

**Table S8 (continued). Summary of previously isolated protolimonoids (Fig. 5*E*) and close derivatives.**

| | | | | | |
|---|---|---|---|---|---|
| 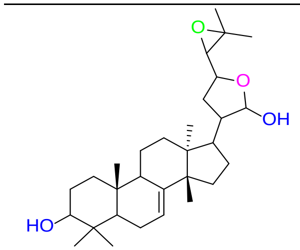 melianol | Meliaceae | Melioideae | *Aglaia odorata* | Stem | 78 |
| | | | *Guarea kunthiana* | Fruit | 70 |
| | | | *Melia azedarach* | | 72 |
| | | | *Melia toosendan* | Fruit | 27 |
| | | | *Trichilia connaroides* | Fruit | 43 |
| | | | *Trichilia hirta* | Fruit | 44 |
| | | | *Quivisia papinae* | Seed | 73 |
| | | Cedreliodeae | *Toona ciliata* | Bark | 57 |
| 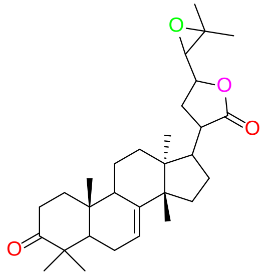 melianone lactone | Meliaceae | Melioideae | *Trichilia hirta* | Fruit | 44 |
| 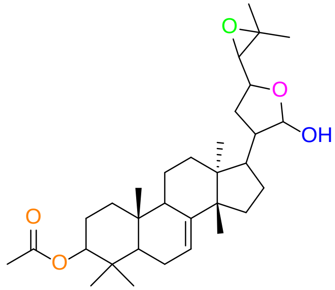 turraeanthin | Meliaceae | Melioideae | *Turraeanthus africanus* | Stem | 79 |

**Table S8 (above). Summary of previously isolated protolimonoids (Fig. 5*E*) and close derivatives.**

Previous isolation of protolimonoids with melianone-, niloticin- or tirucalla-7,24-dien-3ß-ol- type structures are listed. The structure of each protolimonoid is given and oxidations are indicated as follows: ketone or aldehyde (red), hydroxylation (blue), epoxidation (green), hemiacetal (pink) and ester (orange). To encompass reported isolation of structures where stereochemistry has not been specified, stereochemistry at C3, C5, C9, C20, C21, C23 and C24 was not specified in searches. The family, subfamily, species and (where possible) tissue of reported isolation are listed, along with the relevant publication.

**Table S9. Ranked list of CYPs biosynthetic gene candidates co-expressed with CsOSC1**

| Citrus microarray probe | *C.sinensis* probe-target (NCBI identifiers) | Given CYP nomenclature | Closest *A.thaliana* CYP homolog | Condition | Rank | Pearson's correlation coefficient |
|---|---|---|---|---|---|---|
| Cit.3915.1.S1_s_at | LOC102620622 XP_006494184.1 | | AtCYP716A1 | Global | 8 | 0.71 |
| | | | | Fruit | 8 | 0.71 |
| | | | | *C.sinensis* | 16 | 0.66 |
| Cit.14829.1.S1_at | LOC102613788 XP_006465005.1 | | AtCYP716A1 | Global | 12 | 0.65 |
| | | | | Fruit | 12 | 0.65 |
| | | | | *C.sinensis* | 23 | 0.69 |
| | | | | Leaf | 69 | 0.77 |
| Cit.35116.1.S1_s_at | LOC102618084 XP_006467299.1 | CsCYP71CD1 | AtCYP71B23 | Leaf | 4 | 0.82 |
| | | | | Global | 17 | 0.60 |
| | | | | Fruit | 54 | 0.54 |
| Cit.11552.1.S1_s_at | LOC102631112. XP_006469495.1 | CsCYP71BQ4 | AtCYP71B23 | Global | 20 | 0.58 |
| | | | | Fruit | 53 | 0.53 |
| | | | | Leaf | 62 | 0.71 |
| Cit.22580.1.S1_at | LOC102629384 XP_006476911.1 | | AtCYP71B24 | Global | 32 | 0.51 |
| | | | | Fruit | 69 | 0.52 |
| Cit.1814.1.S1_s_at | LOC102615599 XP_006474919.1 | | AtCYP71B23 | *C.sinensis* | 25 | 0.57 |
| | | | | Stress | 62 | 0.68 |
| Cit.28253.1.S1_at | LOC102609586 XP_006485812.1 | | AtCYP71B3 | *C.sinensis* | 99 | 0.60 |
| Cit.11242.1.S1_at | LOC102621266 XP_006493374.1 | | AtCYP76C1 | Leaf | 82 | 0.79 |

**Table S9 (above). Ranked list of CYPs biosynthetic gene candidates co-expressed with CsOSC1**

List of *C. sinensis* CYPs biosynthetic gene candidates co-expressed with CsOSC1 (microarray probe: Cit.36950.1.S1_s_at) across 297 publicly available Citrus genome array datasets from 19 Citrus experiments. Candidate list was generated using the Network Interference for Citrus Co-Expression (NICCE: http://citrus.adelaide.edu.au/nicce/home.aspx). Ranked Pearson's correlation coefficient between CsOSC1 and various CYP genes are shown across datasets grouped by condition-independent datasets (global) and condition-dependent datasets (*C.sinensis*, fruit, leaf, stress). Only CYP genes ranked among the top 100 genes are included in this list. Co-expression information for CYP71CD1 and CYP71BQ4, enzymes characterised in this study (Fig. 5D), is highlighted in grey.

**Table S10. Primers**

| | Primer | Sequence | Target |
|---|---|---|---|
| **OSC candidate yeast recombination primers** | | | |
| 1 | AiOSC1_pYES2-F | ACTACTAGCAGCTGTAATACGACTCACTATAGGGAATATTAATGTGGAAGCTGAAGATTG | *AiOSC1* frag1. |
| 2 | AiOSC1_join-R | TTCAGGATCCTCCACCCAACAAGCAAGCATACACAGC | *AiOSC1* frag1. |
| 3 | AiOSC1_join-F | GCTGTGTATGCTTGCTTGTTGGGTGGAGGATCCTGAA | *AiOSC1* frag2. |
| 4 | AiOSC1_pYES2-R | GAATGTAAGCGTGACATAACTAATTACATGATGCGGCCCTTTAATTAGGCAATGGAAC | *AiOSC1* frag2., *MaOSC1* |
| 5 | MaAZA1_pYES2-F | ACTACTAGCAGCTGTAATACGACTCACTATAGGGAATATTAATGTGGAAGCTGAAGGTTGCAGAG | *MaOSC1* |
| 6 | CsOSC1_PYES2-F | ACTACTAGCAGCTGTAATACGACTCACTATAGGGAATATTAATGTGGAGGCTGAAGGTTGC | *CsOSC1* frag1. |
| 7 | CsOSC1_join-R | CGTTTGGATCTTCAACCCAACAAGCAAGCATACACAACG | *CsOSC1* frag1. |
| 8 | CsOSC1_join-F | CGTTGTGTATGCTTGCTTGTTGGGTTGAAGATCCAAACG | *CsOSC1* frag2. |
| 9 | CsOSC1_PYES2-R | GAATGTAAGCGTGACATAACTAATTACATGATGCGGCCCTTTAATTAGGCAGTGGAACTC | *CsOSC1* frag2. |
| 10 | CsOSC2_PYES2-F | ACTACTAGCAGCTGTAATACGACTCACTATAGGGAATATTAATGTGGAAGCTAAAGGTAGGAG | *CsOSC2* frag1. |
| 11 | CsOSC2_join-R | CTTCCAAAGCTCTGCATCTTCATTCCATCCTCAGCAACCC | *CsOSC2* frag1. |
| 12 | CsOSC2_join-F | GGGTTGCTGAGGATGGAATGAAGATGCAGAGCTTTGGAAG | *CsOSC2* frag2. |
| 13 | CsOSC2_PYES2-R | GAATGTAAGCGTGACATAACTAATTACATGATGCGGCCCTTCAAGAAAGAGTAACCTGATG | *CsOSC2* frag2. |
| 14 | CsOSC3_PYES2-F | ACTACTAGCAGCTGTAATACGACTCACTATAGGGAATATTAATGTGGAGGCTTAAGATTGG | *CsOSC3* frag1. |
| 15 | CsOSC3_join-R | CCATTAGGATCTTCCGCCCAACAGGAGAGCATGTTCAGCG | *CsOSC3* frag1. |
| 16 | CsOSC3_join-F | CGCTGAACATGCTCTCCTGTTGGGCGGAAGATCCTAATGG | *CsOSC3* frag2. |
| 17 | CsOSC3_PYES2-R | GAATGTAAGCGTGACATAACTAATTACATGATGCGGCCCTTCAGAAAATCTTGGACGATTG | *CsOSC3* frag2. |
| 18 | AtLUP5_PYES2-F | ACTACTAGCAGCTGTAATACGACTCACTATAGGGAATATTAATGTGGAGGTTAAAGGTAG | *AtLUP5* |
| 19 | AtLUP5_PYES2-R | GAATGTAAGCGTGACATAACTAATTACATGATGCGGCCCTCTATAGATCTGCGTGATGT | *AtLUP5* |

**Table S10 (continued). Primers**

| | | | |
|---|---|---|---|
| ***OSC candidate gateway cloning*** | | | |
| 20 | AiOSC1_AttB1-F | GGGGACAAGTTTGTACAAAAAAGCAGGCTTCATGTGGAAGCTGAAGATTG | *AiOSC1* |
| 21 | AiOSC1_AttB2-R | GGGGACCACTTTGTACAAGAAAGCTGGGTTTTAATTAGGCAATGGAAC | *AiOSC1,MaOSC1* |
| 22 | MaOSC1_AttB1-F | GGGGACAAGTTTGTACAAAAAAGCAGGCTTCATGTGGAAGCTGAAGGTTGCAGAGGG | *MaOSC1* |
| 23 | CsOSC1_AttB1-F | GGGGACAAGTTTGTACAAAAAAGCAGGCTTCAATGTGGAGGCTGAAGGTTGC | *CsOSC1* |
| 24 | CsOSC1_AttB2-R | GGGGACCACTTTGTACAAGAAAGCTGGGTTTTAATTAGGCAGTGGAACTC | *CsOSC1* |
| 25 | CsOSC2_AttB1-F | GGGGACAAGTTTGTACAAAAAAGCAGGCTTCATGTGGAAGCTAAAGGTAGGAG | *CsOSC2* |
| 26 | CsOSC2_AttB2-R | GGGGACCACTTTGTACAAGAAAGCTGGGTTTCAAGAAAGAGTAACCTGATG | *CsOSC2* |
| 27 | CsOSC3_AttB1-F | GGGGACAAGTTTGTACAAAAAAGCAGGCTTCATGTGGAGGCTTAAGATTGG | *CsOSC3* |
| 28 | CsOSC3_AttB2-R | GGGGACCACTTTGTACAAGAAAGCTGGGTTTTCAGAAAATCTTGGACGATTG | *CsOSC3* |
| ***Sequencing- plasmid specific*** | | | |
| 29 | GAL1-F | AATATACCTCTATACTTTAACGTC | *pYES2* |
| 30 | pYES2-R | GCGTGAATGTAAGCGTGAC | *pYES2* |
| 31 | AttL1-F | TCGCGTTAACGCTAGCAT | *pDNR207* |
| 32 | AttL2-R | GTAACATCAGAGATTTTGAGACAC | *pDNR207* |
| 33 | AttB1-F | GGGGACAAGTTTGTACAAAAAAGCAGGCTTA | *pEAQ-HT-DEST1, pYES2-DEST52, pAG423GAL, pAG425GAL* |
| 34 | AttB2-R | GGGGACCACTTTGTACAAGAAAGCTGGGTA | *pEAQ-HT-DEST1, pYES2-DEST52, pAG423GAL, pAG425GAL* |
| ***Sequencing- sequence specific*** | | | |
| 35 | AiOSC1_middle-F | GGAAAGAATTGGCTTTCG | *AiOSC1,MaOSC1* |
| 36 | CsOSC1_middle-F | TGGGGAAAGAATTGGCTTTC | *CsOSC1* |
| 37 | CsOSC2_middle-F | CTTCCTGGGGAAAGACTTGG | *CsOSC2* |
| 38 | CsOSC3_middle-F | CATCTTGGGGAAAGACTTGG | *CsOSC3* |

**Table S10 (continued). Primers**

| | | | |
|---|---|---|---|
| ***CYP candidate gateway cloning*** | | | |
| 39 | MaCYP72A720_AttB1-F | GGGGACAAGTTTGTACAAAAAAGCAGGCTTCATGGAGTTATCTCTGAAATCGG | *MaCYP72A720* |
| 40 | MaCYP72A720_AttB1-R | GGGGACCACTTTGTACAAGAAAGCTGGGTTTTATAATTTCTTTAAAATCAAG | *MaCYP72A720* |
| 41 | MaCYP71BQ5_AttB1-F | GGGGACAAGTTTGTACAAAAAAGCAGGCTTCATGGAGTTCAGACTGCCTGTTC | *MaCYP71BQ5* |
| 42 | MaCYP71BQ5_AttB1-R | GGGGACCACTTTGTACAAGAAAGCTGGGTTTCACTTCTGAAAAGGAATACGAG | *MaCYP71BQ5* |
| 43 | MaCYP88A108_AttB1-F | GGGGACAAGTTTGTACAAAAAAGCAGGCTTCATGGAGCTAAATTTCCTGTGG | *MaCYP88A108* |
| 44 | MaCYP88A108_AttB1-R | GGGGACCACTTTGTACAAGAAAGCTGGGTTTCAGAAGTTCTTGACCTTGATG | *MaCYP88A108* |
| 45 | MaCYP71BQ6_AttB1-F | GGGGACAAGTTTGTACAAAAAAGCAGGCTTAATGGATTCTTCAATATCCC | *MaCYP71BQ6* |
| 46 | MaCYP71BQ6_AttB2-R | GGGGACCACTTTGTACAAGAAAGCTGGGTATCACTTCTGTAAAGGAAAACG | *MaCYP71BQ6* |
| 47 | MaCYP71D557_AttB1-F | GGGGACAAGTTTGTACAAAAAAGCAGGCTTAATGGAGGTTCAATTTGTTTCC | *MaCYP71D557* |
| 48 | MaCYP71D557_AttB2-R | GGGGACCACTTTGTACAAGAAAGCTGGGTATCATGGATGATCAGCCGG | *MaCYP71D557* |
| 49 | MaCYP71CD2_AttB1-F | GGGGACAAGTTTGTACAAAAAAGCAGGCTTAATGAATCTCCAACTCGATTAC | *MaCYP71CD* |
| 50 | MaCYP71CD2_AttB2-R | GGGGACCACTTTGTACAAGAAAGCTGGGTATTAATTTTCCACCTCAATGTTG | *MaCYP71CD* |
| 51 | MaCYP71BE124_AttB1F | GGGGACAAGTTTGTACAAAAAAGCAGGCTTAATGGAGTACCAACTTCCATC | *MaCYP71BE124* |
| 52 | MaCYP71BE124_AttB2R | GGGGACCACTTTGTACAAGAAAGCTGGGTACTAGACTAGTTTGGAGTTATTG | *MaCYP71BE124* |
| ***CYP candidate Gibson cloning*** | | | |
| 53 | CsCYP71CD1-F | ATTCTGCCCAAATTCGCGACCGGTATGGAGCAACAATTTGATTACTTCACTG | *CsCYP71CD1* |
| 54 | CsCYP71CD1-R | GAAACCAGAGTTAAAGGCCTCGAGTCACGGAATATTATTGTAAGGAGTAGG | *CsCYP71CD1* |
| 55 | CsCYP71BQ4-F | ATTCTGCCCAAATTCGCGACCGGTATGGACATTACTACTACAGCAACC | *CsCYP71BQ4* |
| 56 | CsCYP71BQ4-R | GAAACCAGAGTTAAAGGCCTCGAGTCACTTCTCAAAAGGAATACGAGTGG | *CsCYP71BQ4* |
| **qRT-PCR** | | | |
| 57 | Maß-actin_qPCR-F | CCAAGCAGCATGAAGATTAAGG | *Maß-actin* |
| 58 | Maß-actin_qPCR-R | ATCTGCTGGAAGGTGCTGAG | *Maß-actin* |
| 59 | MaOSC1_qPCR-F | GTTGAGCATACTTATGTGGAATGC | *MaOSC1* |
| 60 | MaOSC1_qPCR-R | CAATCTCCTTCTTGCGATGATGTGGG | *MaOSC1* |
| 61 | MaCYP71CD2_qPCR-F | TGTGATTTGGAGTGTATTTGC | *MaCYP71CD* |

| | | | |
|---|---|---|---|
| 62 | MaCYP71CD2_qPCR-F | GGATTTCTCATCAATTCTGAC | *MaCYP71CD* |
| 63 | MaCYP71BQ5_qPCR-F | CTGTCACTCTGGACATTTTCTCAG | *MaCYP71BQ5* |
| 64 | MaCYP71BQ5_qPCR-R | GCTTGTCGGACCTCTTCTTGTGCC | *MaCYP71BQ5* |

The nucleotide sequences of all primers used and their target genes are listed including, those used for cloning, sequencing and qRT-PCR.

**Table S11. Isolera™ Prime fractionation conditions.**

| Target | Column | Solvents | Gradient | Yield |
|---|---|---|---|---|
| *AiOSC1* product | SNAP Ultra 10g | A: Hexane<br>B: EtOAc | 0-2%(1CV), 2-25% (10CV), 25-100% (3CV) | 1 mg |
| *AiOSC1* *MaCYP71BQ5* product | KP-Sil 50g | A:Hexane<br>B: EtOAc | 0-100%(45CV), 100%(11CV) | 50 mg |
| | SNAP Ultra 10g | A:Hexane<br>B: EtOAc | 20-50% (176CV),50%(5CV) | 10 mg |
| | SNAP Ultra 10g | A:Hexane<br>B: EtOAc | 20%(176CV) | 4 mg |
| *AiOSC1* *MaCYP71CD2* product | SNAP KP-Sil 25g | A:Hexane<br>B: EtOAc | 6-100% (10CV) | 190 mg |
| | SNAP Ultra 10g | A:Hexane<br>B: EtOAc | 10%(1CV), 10-60%(10CV), 60%(5CV) | 170 mg |
| | SNAP Ultra 10g | A:Hexane<br>B: EtOAc | 10% (3CV), 10-34% (143CV) | 130 mg |
| | SNAP Ultra 10g | A:Hexane<br>B: EtOAc | 0-30%(181CV) | 120 mg |
| *AiOSC1* *MaCYP71CD2* *MaCYP71BQ5* product | SNAP KP-Sil 100g | A:Hexane<br>B: EtOAc | 6-100% (13CV) | 410 mg |
| | SNAP Ultra 10g | A:Hexane<br>B: EtOAc | 20-53% (116CV) | 220 mg |
| | SNAP Ultra 10g | A: Hexane<br>B:DCM | 10-41% (140CV) | 34 mg |
| | SNAP Ultra 10g | A: Hexane<br>B:DCM | 10-30% (87CV), 30% (79CV) | 4 mg |

Details of conditions used for Isolera™ Prime fractionation including: column, solvent system, percentage gradient of solvent B, column volume and dry weight of resulting extract (yield). All samples were dry-loaded onto Isolera™ Prime (Biotage) using Celite® (Sigma-Aldrich).

**Table S12. Media used for *S. cerevisiae* selection**

| **Plasmid** | **Selection** | **Medium** |
|---|---|---|
| pYES2 | URA3 | SD-URA |
| pAG423GAL | HIS3 | SD-HIS |
| pAG425GAL | LEU2 | SD-LEU |
| pYES2,<br>pAG423GAL,<br>pAG425GAL | URA3<br>HIS3<br>LEU2 | SD-URA -HIS -LEU |

Supplement drop-out (SD) media used to select for each *S. cerevisiae* plasmid. Selection amino are include uracil (URA), histidine (HIS) and leucine (LEU).

## References


1. MacKenzie DJ, McLean MA, Mukerji S, & Green M (1997) Improved RNA extraction from woody plants for the detection of viral pathogens by reverse transcription-polymerase chain reaction. *Plant Disease* 81(2):222-226.
2. Kushiro T, Shibuya M, Masuda K, & Ebizuka Y (2000) Mutational studies on triterpene synthases: engineering lupeol synthase into β-amyrin synthase. *Journal of the American Chemical Society* 122(29):6816-6824.
3. Sainsbury F, Thuenemann EC, & Lomonossoff GP (2009) pEAQ: versatile expression vectors for easy and quick transient expression of heterologous proteins in plants. *Plant Biotechnology Journal* 7(7):682-693.
4. Reed J*, et al.* (2017) A translational synthetic biology platform for rapid access to gram-scale quantities of novel drug-like molecules. *Metabolic Engineering* 42:185-193.
5. Krishnan NM*, et al.* (2011) De novo sequencing and assembly *of Azadirachta indica* fruit transcriptome. *Current Science* 101(12):1553-1561.
6. Krishnan NM*, et al.* (2012) A draft of the genome and four transcriptomes of a medicinal and pesticidal angiosperm *Azadirachta indica*. *BMC Genomics* 13(1):464.
7. Haas BJ*, et al.* (2013) De novo transcript sequence reconstruction from RNA-seq using the Trinity platform for reference generation and analysis. *Nature Protocols* 8(8):1494-1512.
8. Langmead B, Trapnell C, Pop M, & Salzberg SL (2009) Ultrafast and memory-efficient alignment of short DNA sequences to the human genome. *Genome Biol* 10(1):R25.
9. Li B & Dewey CN (2011) RSEM: accurate transcript quantification from RNA-Seq data with or without a reference genome. *BMC Bioinformatics* 12(1):323.

10. Robinson M, McCarthy D, & Smyth G (2010) edgeR: a Bioconductor package for differential expression analysis of digital gene expression data. *Bioinformatics* 26(1):139-140.
11. Love M, Huber W, & Anders S (2014) Moderated estimation of fold change and dispersion for RNA-seq data with DESeq2. *Genome Biology* 15(12):550.
12. Anonymous (2008) Spearman rank correlation coefficient. The concise encyclopedia of statistics, eds Springer New York, (New York, NY), pp 502-505.
13. Pearson K (1895) Notes on regression and inheritance in the case of two parents. *Proceedings of the Royal Society of London*, 58(1): 240-242.
14. Zhao S, Guo Y, Sheng Q, & Shyr Y (2014) Heatmap3: an improved heatmap package with more powerful and convenient features. *BMC Bioinformatics* 15(10):P16.
15. Pandreka A*, et al.* (2015) Triterpenoid profiling and functional characterization of the initial genes involved in isoprenoid biosynthesis in neem (*Azadirachta indica*). *BMC Plant Biology* 15:14.
16. Stephenson MJ, Reed J, Brouwer B, & Osbourn A (2018) Transient expression in *Nicotiana benthamiana* leaves for triterpene production at a preparative scale. *JoVE* 138(1):e58169.
17. Bak S*, et al.* (2011) Cytochromes P450. *The Arabidopsis Book* 9(1):e0144-e0144.
18. Price M, Dehal P, & Arkin A (2010) FastTree 2-approximately maximum-likelihood trees for large alignments. *PLOS ONE* 5(3): e9490.
19. Letunic I & Bork P (2016) Interactive tree of life (iTOL) v3: an online tool for the display and annotation of phylogenetic and other trees. *Nucleic Acids Research* 44(W1):W242-W245.
20. Sievers F*, et al.* (2011) Fast, scalable generation of high-quality protein multiple sequence alignments using Clustal Omega. *Molecular Systems Biology* 7(1):539.

21. Wang Y*, et al.* (2016) Comparative analysis of the terpenoid biosynthesis pathway in Azadirachta indica and *Melia azedarach* by RNA-seq. *SpringerPlus* 5(1):1-9.
22. Ebizuka Y, Katsube Y, Tsutsumi T, Kushiro T, & Shibuya M (2003) Functional genomics approach to the study of triterpene biosynthesis. *Pure and Applied Chemistry*, p 369.
23. Morlacchi P*, et al.* (2009) Product profile of PEN3: the last unexamined oxidosqualene cyclase in *Arabidopsis thaliana*. *Organic Letters* 11(12):2627-2630.
24. Nelson DR (2006) Cytochrome P450 nomenclature, 2004. *Cytochrome P450 Protocols*, eds Phillips IR & Shephard EA (Humana Press, Totowa, NJ), pp 1-10.
25. Mittapelli SR, Maryada SK, Khareedu VR, & Vudem DR (2014) Structural organization, classification and phylogenetic relationship of cytochrome P450 genes in *Citrus clementina* and *Citrus sinensis*. *Tree Genetics & Genomes* 10(2):399-409.
26. Gray AI, Bhandari P, & Waterman PG (1988) New protolimonoids from the fruits of *Phellodendron chinense*. *Phytochemistry* 27(6):1805-1808.
27. Nakanishi T, Inada A, & Lavie D (1986) A new tirucallane-type triterpenoid derivative, lipomelianol from fruits of *Melia toosendan* Sieb. et Zucc. *Chemical and Pharmaceutical Bulletin* 34(1):100-104.
28. Zhao W-Y*, et al.* (2019) New tirucallane triterpenoids from *Picrasma quassioides* with their potential antiproliferative activities on hepatoma cells. *Bioorganic Chemistry* 84(1):309-318.
29. Mulholland DA, Kotsos M, Mahomed HA, & Taylor DAH (1998) Triterpenoids from *Owenia cepiodora*. *Phytochemistry* 49(8):2457-2460.
30. Wattanapiromsakul C & Waterman PG (2000) Flavanone, triterpene and chromene derivatives from the stems of *Paramignya griffithii*. *Phytochemistry* 55(3):269-273.

31. Fo ER, Fernandes JB, Vieira PC, & Da Silva MFDGF (1992) Isolation of secoisolariciresinol diesters from stems of *Simaba cuneata*. *Phytochemistry* 31(6):2115-2116.

32. Polonsky J, Baskevitch-Varon Z, & Das BC (1976) Triterpenes tetracycliques du *Simarouba amara*. *Phytochemistry* 15(2):337-339.

33. Luo X-D, Wu S-H, Ma Y-B, & Wu D-G (2000) Tirucallane triterpenoids from *Dysoxylum hainanense*. *Phytochemistry* 54(8):801-805.

34. Liu H, Heilmann J, Rali T, & Sticher O (2001) New tirucallane-type triterpenes from *Dysoxylum variabile*. *Journal of Natural Products* 64(2):159-163.

35. Kumar V, Niyaz NMM, Wickramaratne DBM, & Balasubramaniam S (1991) Tirucallane derivatives from *Paramignya monophylla* fruits. *Phytochemistry* 30(4):1231-1233.

36. Jayakumar G, Ajitha Bai MD, & Fujimoto Y (2004) Beddomeilactone: a new triterpene from *Dysoxylum Beddomei* AU - Hisham, A. *Natural Product Research* 18(4):329-334.

37. Gu J*, et al.* (2013) Chemical components of *Dysoxylum densiflorum*. *Natural Products and Bioprospecting* 3(2):66-69.

38. Grosvenor SNJ, Mascoll K, McLean S, Reynolds WF, & Tinto WF (2006) Tirucallane, apotirucallane, and octanorapotirucallane triterpenes of *Simarouba amara*. *Journal of Natural Products* 69(9):1315-1318.

39. Mohamad K*, et al.* (1999) Tirucallane triterpenes from *Dysoxylum macranthum*. *Phytochemistry* 52(8):1461-1468.

40. Orisadipe AT, Adesomoju AA, D'Ambrosio M, Guerriero A, & Okogun JI (2005) Tirucallane triterpenes from the leaf extract of *Entandrophragma angolense*. *Phytochemistry* 66(19):2324-2328.

41. Chen J*, et al.* (2011) Cytotoxic triterpenoids from *Azadirachta indica*. *Planta medica* 77(16):1844-1847.

42. Ragasa CY*, et al.* (2013) Glabretal-type triterpenoids from *Dysoxylum mollissimum*. *Phytochemistry Letters* 6(4):514-518.

43. Inada A, Konishi M, Murata H, & Nakanishi T (1994) Structures of a new limonoid and a new triterpenoid derivative from pericarps of *Trichilia connaroides. Journal of Natural Products* 57(10):1446-1449.
44. Vieira JI*, et al.* (2013) Hirtinone, a novel cycloartane-type triterpene and other compounds from *Trichilia hirta* L. (Meliaceae). *Molecules* 18(3):2589-2597.
45. Rodrigues VF, Carmo HM, Braz RF, Mathias L, & Vieira I (2010) Two new terpenoids from *Trichilia quadrijuga* (Meliaceae). *Natural Product Communications* 5(2):179-184.
46. Harding WW, Jacobs H, Lewis PA, McLean S, & Reynolds WF (2001) Cycloartanes, protolimonoids, a pregnane and a new ergostane from *Trichilia reticulata*. *Natural Product Letters* 15(4):253-260.
47. Ketwaru P, Klass J, Tinto WF, McLean S, & Reynolds WF (1993) Pregnane steroids from *Trichilia schomburgkii*. *Journal of Natural Products* 56(3):430-431.
48. Tinto WF, Jagessar PK, Ketwaru P, Reynolds WF, & McLean S (1991) Constituents of *Trichilia schomburgkii*. *Journal of Natural Products* 54(4):972-977.
49. Wang G-C*, et al.* (2016) Limonoids and triterpenoids as 11β-HSD1 inhibitors from *Walsura robusta*. *Journal of Natural Products* 79(4):899-906.
50. Liu J-Q*, et al.* (2012) Limonoids from the leaves of *Toona ciliata* var. yunnanensis. *Phytochemistry* 76(1):141-149.
51. Kishi K, Yoshikawa K, & Arihara S (1992) Limonoids and protolimonoids from the fruits of *Phellodendron amurense*. *Phytochemistry* 31(4):1335-1338.
52. Itokawa H, Kishi E, Morita H, & Takeya K (1992) Cytotoxic quassinoids and tirucallane-type triterpenes from the woods of *Eurycoma longifolia. Chemical & Pharmaceutical Bulletin* 40(4):1053-1055.
53. Saraiva RdCG, Pinto AC, Nunomura SM, & Pohlit AM (2006) Triterpenes and a canthinone alkaloid from the stems of *Simaba polyphylla*

(Cavalcante) WW Thomas (Simaroubaceae). *Química Nova* 29(2):264-268.

54. Esimone CO*, et al.* (2008) Potential anti-respiratory syncytial virus lead compounds from *Aglaia species*. *Die Pharmazie - An International Journal of Pharmaceutical Sciences* 63(10):768-773.

55. Benosman A*, et al.* (1995) Tirucallane triterpenes from the stem bark of *Aglaia leucophylla*. *Phytochemistry* 40(5):1485-1487.

56. Irungu BN*, et al.* (2015) Antiplasmodial and cytotoxic activities of the constituents of *Turraea robusta* and *Turraea nilotica*. *Journal of Ethnopharmacology* 174:419-425.

57. Wang J-R*, et al.* (2011) Protolimonoids and norlimonoids from the stem bark of *Toona ciliata* var. pubescens. *Organic & Biomolecular Chemistry* 9(22):7685-7696.

58. Ahsan M, Armstrong JA, Gray AI, & Waterman PG (1994) Boronialatenolide: a novel pentanortriterpene from the aerial parts of *Boronia alata* (Rutaceae). *Australian Journal of Chemistry* 47(9):1783-1787.

59. Ahsan M, Armstrong JA, Gray AI, & Waterman PG (1995) Terpenoids, alkaloids and coumarins from *Boronia inornata* and *Boronia gracilipes*. *Phytochemistry* 38(5):1275-1278.

60. Reegan AD, Gandhi MR, Paulraj MG, Balakrishna K, & Ignacimuthu S (2014) Effect of niloticin, a protolimonoid isolated from *Limonia acidissima* L. (Rutaceae) on the immature stages of dengue vector *Aedes aegypt*i L. (Diptera: Culicidae). *Acta Tropica* 139(1):67-76.

61. Lien TP, Kamperdick C, Schmidt J, Adam G, & Van Sung T (2002) Apotirucallane triterpenoids from *Luvunga sarmentosa* (Rutaceae). *Phytochemistry* 60(7):747-754.

62. Kiplimo J, Islam S, & Koorbanally N (2012) Ring A, D-SECO limonoids and flavonoid from the Kenyan *Vepris uguenensis* Engl. and their antioxidant activity. *Planta Medica* 78(11):PI111.

63. Hong Z-L*, et al.* (2013) Tetracyclic triterpenoids and terpenylated coumarins from the bark of *Ailanthus altissima* ("tree of heaven"). *Phytochemistry* 86(1):159-167.

64. Grieco PA, Haddad J, Piñeiro-Núñez MM, & Huffman JC (1999) Quassinoids from the twigs and thorns of *Castela polyandra*. *Phytochemistry* 50(4):637-645.

65. Wang J, Zhang Y, Luo J, & Kong L (2011) Complete 1H and 13C NMR data assignment of protolimonoids from the stem barks of *Aphanamixis grandifolia*. *Magnetic Resonance in Chemistry* 49(7):450-457.

66. Zhang X-Y*, et al.* (2010) Tirucallane-type alkaloids from the bark of *Dysoxylum laxiracemosum*. *Journal of Natural Products* 73(8):1385-1388.

67. Huang HL*, et al.* (2011) Tirucallane-type triterpenoids from *Dysoxylum lenticellatum. Journal of Natural Products* 74(10):2235-2242.

68. Hayasida W, Oliveira L, Ferreira A, & Lima M (2017) Ergostane steroids, tirucallane and apotirucallane triterpenes from *Guarea convergens*. *Chemistry of Natural Compounds* 53(2):312-317.

69. Jimenez A*, et al.* (1998) Limonoids from *Swietenia humilis* and *Guarea grandiflora* (Meliaceae) Taken in part from the PhD and MS theses of C. Villarreal and M. A. Jiménez, respectively. *Phytochemistry* 49(7):1981-1988.

70. Miguita CH*, et al.* (2015) 3β-O-tigloylmelianol from *Guarea kunthiana*: a new potential agent to control rhipicephalus (boophilus) microplus, a cattle tick of veterinary significance. *Molecules* 20(1):111.

71. Ntalli NG*, et al.* (2010) Cytotoxic tirucallane triterpenoids from *Melia azedarach* fruits. *Molecules* 15(9):5866-5877.

72. Han J, Lin W, Xu R, Wang W, & Zhao S (1991) Studies on the chemical constituents of *Melia azedarach* L. *Acta pharmaceutica Sinica* 26(6):426-429.

73. Coombes PH, Mulholland DA, & Randrianarivelojosia M (2005) Mexicanolide limonoids from the Madagascan Meliaceae *Quivisia papinae*. *Phytochemistry* 66(10):1100-1107.

74. Kaur R & Arora S (2009) Chemical constituents and biological activities of *Chukrasia tabularis* A. Juss.-A review. *Journal of Medicinal Plants Research* 3(4):196-216.
75. Basak S & Islam A (1970) DP Melianone from *Swietenia mahagoni*. J. *Indian Chem. Soc.* 47(5):501-503.
76. Su R*, et al.* (1990) Triterpenoids from the fruits of *Phellodendron chinense*: The stereostructure of niloticin. *Chemical and Pharmaceutical Bulletin* 38(6):1616-1619.
77. Biavatti MW*, et al.* (2001) Chemistry and bioactivity of *Raulinoa echinata* Cowan, an endemic Brazilian Rutaceae species. *Phytomedicine* 8(2):121-124.
78. Yang S-P, Ni G, Gu Y-C, & Yue J-M (2012) Triterpenoids from *Aglaia odorata* var. microphyllina AU - Liu, Jia. *J. Asian Nat. Prod. Res.* 14(10):929-939.
79. Bevan C, Ekong D, Halsall T, & Toft P (1967) West African timbers. Part XX. The structure of turraeanthin, an oxygenated tetracyclic triterpene monoacetate. *Journal of the Chemical Society C: Organic*:820-828.
